# Discovery of novel amino acid production traits by evolution of synthetic co-cultures

**DOI:** 10.1186/s12934-023-02078-2

**Published:** 2023-04-15

**Authors:** Rico Zuchowski, Simone Schito, Friederike Neuheuser, Philipp Menke, Daniel Berger, Niels Hollmann, Srushti Gujar, Lea Sundermeyer, Christina Mack, Astrid Wirtz, Oliver H. Weiergräber, Tino Polen, Michael Bott, Stephan Noack, Meike Baumgart

**Affiliations:** 1 Institute of Bio- and Geosciences, IBG-1: Biotechnology, Forschungszentrum Jülich, Jülich, Germany; 2 Institute of Biological Information Processing, IBI-7: Structural Biochemistry, Forschungszentrum Jülich, Jülich, Germany; 3 Institut für Physikalische Biologie, Heinrich-Heine-Universität Düsseldorf, Düsseldorf, Germany

**Keywords:** Synthetic co-culture, *Corynebacterium glutamicum*, ALE / evolutionary engineering, Arginine import, Metabolic engineering, Arginine production

## Abstract

**Background:**

Amino acid production features of *Corynebacterium glutamicum* were extensively studied in the last two decades. Many metabolic pathways, regulatory and transport principles are known, but purely rational approaches often provide only limited progress in production optimization. We recently generated stable synthetic co-cultures, termed Communities of Niche-optimized Strains (CoNoS), that rely on cross-feeding of amino acids for growth. This setup has the potential to evolve strains with improved production by selection of faster growing communities.

**Results:**

Here we performed adaptive laboratory evolution (ALE) with a CoNoS to identify mutations that are relevant for amino acid production both in mono- and co-cultures. During ALE with the CoNoS composed of strains auxotrophic for either l-leucine or l-arginine, we obtained a 23% growth rate increase. Via whole-genome sequencing and reverse engineering, we identified several mutations involved in amino acid transport that are beneficial for CoNoS growth. The l-leucine auxotrophic strain carried an expression-promoting mutation in the promoter region of *brnQ* (cg2537), encoding a branched-chain amino acid transporter in combination with mutations in the genes for the Na^+^/H^+^-antiporter Mrp1 (cg0326-cg0321). This suggested an unexpected link of Mrp1 to l-leucine transport. The l-arginine auxotrophic partner evolved expression-promoting mutations near the transcriptional start site of the yet uncharacterized operon *argTUV* (cg1504-02). By mutation studies and ITC, we characterized ArgTUV as the only l-arginine uptake system of *C. glutamicum* with an affinity of K_D_ = 30 nM. Finally, deletion of *argTUV* in an l-arginine producer strain resulted in a faster and 24% higher l-arginine production in comparison to the parental strain.

**Conclusion:**

Our work demonstrates the power of the CoNoS-approach for evolution-guided identification of non-obvious production traits, which can also advance amino acid production in monocultures. Further rounds of evolution with import-optimized strains can potentially reveal beneficial mutations also in metabolic pathway enzymes. The approach can easily be extended to all kinds of metabolite cross-feeding pairings of different organisms or different strains of the same organism, thereby enabling the identification of relevant transport systems and other favorable mutations.

**Supplementary Information:**

The online version contains supplementary material available at 10.1186/s12934-023-02078-2.

## Background

In nature, microorganisms usually live in communities with various other organisms leading to a large number of intraspecies and interspecies interactions. Some organisms are competitors e.g. for nutrients, but at the same time there are numerous cooperations that have beneficial effects for all community members. These kind of syntrophic interactions are a key factor in species evolution and can enable bacteria to use resources more efficiently and grow with a higher productivity [1]. Metabolic auxotrophies can be advantageous for the cells, because uptake of a certain metabolite from the environment is usually cheaper than synthesizing it itself [2]. In this way, whole communities adapt to very specific ecological niches in which the supply with metabolites occurs by co-evolving partners [3].

Based on these observations, we asked the question whether it is possible to generate synthetic communities that can produce a compound of interest more efficiently than a monoculture and developed the concept of Communities of Niche-optimized Strains, in short CoNoS [3]. A CoNoS consists of at least two strains of the same species that are auxotrophic for a certain metabolite and cross-feed each other with the aim to use the available resources more efficiently than a monoculture. In this context, former studies already showed that synthetic communities comprising auxotrophic *Escherichia* *coli* strains reached a higher biomass and fitness level compared to the monocultures [4, 5]. Furthermore, initial communities with sub-optimal interactions evolved quickly and both partners improved their metabolite production to benefit their corresponding partner [6].

*Corynebacterium glutamicum* is one of the most important workhorses of industrial biotechnology for the production of amino acids and various other metabolites [7, 8]. Many metabolic pathways as well as regulatory and transport principles are known, but further improvements of amino acid production strains by rational approaches became less and less in the last years. Thus, we decided to use *C. glutamicum* as model organism for our CoNoS approach [9]. We established a fast and stable growing community of an l-arginine and an l-leucine auxotrophic strain, both rationally engineered for higher production of the amino acid required by their partner strain. This CoNoS reached a growth rate equivalent to 83% of the wild type, suggesting some remaining bottlenecks in their cross-feeding relationship [9]. These bottlenecks are most likely related either to amino acid production, which is well studied in *C. glutamicum*, or to transport processes. Amino acid export [10] and import (Additional file 1: Table S1) is known to some extent in *C.* *glutamicum*, but there are still several gaps, e.g. it is unclear how l-arginine is taken up by the cell. This makes rational approaches rather difficult. One important alternative is the untargeted approach of Adaptive Laboratory Evolution (ALE), which exploits the natural principle of evolutionary adaptation of cells to changing environmental conditions. Many different strategies and technologies have been established to perform ALE experiments [11, 12] and in particular the repetitive batch approach has gained popularity due to its low operating costs, simple experimental implementation, and easy extensibility [13]. In combination with liquid handling robotics, automated ALE experiments can be performed, leading to standardized and robust procedures and thus increasing the chances of successful evolution with identification of new strain features relevant for production [14, 15].

In this study, we performed automated ALE with a CoNoS to identify mutations beneficial for amino acid production and transport both in synthetic communities and monocultures. We isolated single strains from faster growing CoNoS and identified several mutations e.g. in promoters of amino acid transport systems. Among others, we identified and characterized ArgTUV as an l-arginine import system. Finally, deletion of this transporter increased the l-arginine titer by 24% for an l-arginine producing monoculture.

## Results

### ALE with a CoNoS comprising two amino acid-auxotrophic strains

**Fig. 1:**
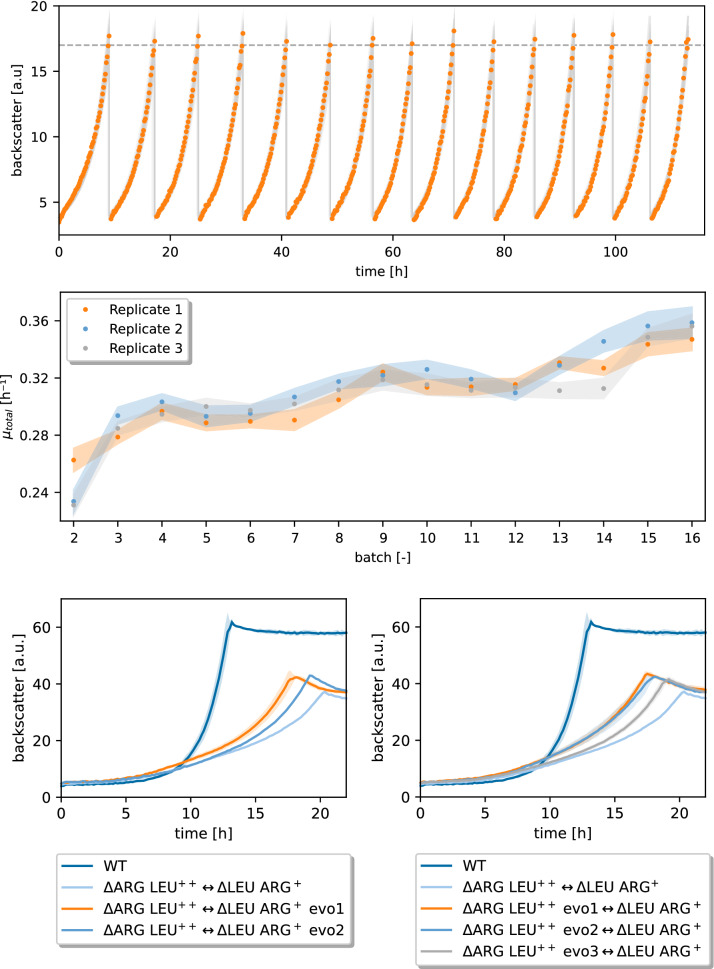
ALE of a CoNoS comprising two amino-acid auxotrophic strains. **A** ALE of the CoNoS composed of *C. glutamicum* ΔARG LEU^++^  ↔ ΔLEU ARG+. To start the ALE, both strains were cultivated in shake flask monocultures in CGXII medium with 2% (w/v) glucose and 3 mM of the required amino acid. After 2 days at 30 °C, the cells were washed and used in a 1:1 ratio based on OD_600_ measurements to inoculate the first batch. 16 repetitive batches were cultivated in CGXII medium with 2% (w/v) glucose and 100 µM IPTG in microtiter plates (MTPs) at 30 °C, 1400 rpm. Displayed is the on-line backscatter signal of batches 2–16. A backscatter threshold (dashed grey line) triggered cell suspension transfer to inoculate the following batch. One representative of three independent replicates is shown. **B** Specific growth rate calculation for the ALE shown in (**A**). **C**, **D** Each evolved strain (evo1, evo2, and evo3) was isolated from one independent replicate of the ALE and cultivated in biological triplicates with the corresponding non-evolved partner. WT monoculture and the CoNoS comprising the non-evolved strains are shown as reference cultivations. The CoNoS were cultivated in CGXII medium with 2% (w/v) glucose and incubated in MTPs at 30 °C, 1400 rpm. Mean values and standard deviations are shown as lines and shaded areas, respectively

We recently demonstrated that synthetic CoNoS composed of two complementary amino acid-auxotrophic strains are able to grow based on mutual dependency [9]. To improve amino acid exchange and thus growth of the CoNoS, the strains were metabolically engineered addressing known targets for increased amino acid production [9]. In this work, we exploit ALE to improve the fitness of microbial communities (Fig. 1A) with the aim to identify and investigate new growth-related targets such as transporters that increase amino acid exchange. As starting point, we chose the l-arginine-auxotrophic strain ΔARG LEU^++^ and the l-leucine-auxotrophic strain ΔLEU ARG^+^. ΔARG LEU^++^ is derived from the *C. glutamicum* wild type lacking all biosynthetic enzymes for l-arginine and carrying a feedback resistant LeuA variant under control of the strong P_*tuf*_ promoter, which leads to a slight overproduction of l-leucine. ΔLEU ARG+ is derived from the genome-reduced strain C1* with an in frame deletion of *argR* (cg1585) and produces a sufficient amount of l-arginine for co-culture growth. A CoNoS composed of these two strains has a growth rate resembling almost half of the WT level, leaving potential for improvement by selecting for faster growing cultures [9].

For the ALE, we used the Mini Pilot Plant that consists of a microbioreactor combined with a liquid handling robotic system [15, 16]. The CoNoS was grown in CGXII medium with 2% (w/v) (corresponding to 111 mM) d-glucose until a backscatter (BS) threshold (BS = 17) triggered automated transfer of 10% (v/v) of the cell suspension into an empty well that was filled with fresh chill-stored CGXII medium. In this manner, we performed 16 repetitive batches in triplicates. Next to online biomass, the time of each transfer was monitored for each single batch, enabling model-based growth rate estimation (Fig. 1B). Both growth rate and transfer time indicated that in all three replicates the fitness of the CoNoS improved across the batches. Specifically, the transfer time decreased by 23% and the growth rate increased by 23% from batch two to batch 16 in all three replicates. At this point, it was not obvious whether the improved fitness was caused by mutations occurring in only one or in both community members and thus the single evolved strains were isolated for further analysis. Cell suspension of the last batch of each replicate was spread onto CGXII agar plates with 2% (w/v) glucose supplemented either with 3 mM l-leucine or 3 mM l-arginine to select only one community member on each plate (six plates in total). Six colonies from each plate were selected and combined in a CoNoS with the corresponding non-evolved partner to test whether the single evolved strains are able to improve growth of a community (Additional file 1: Fig. S1). Interestingly, not all clones from one ALE affected growth of the CoNoS similarly, which means that even after 16 batches there is obvious heterogeneity in the cultures (Additional file 1: Fig. S1). The best performing clone from each plate was selected for further characterization and named ∆LEU ARG^+^ evo1, ∆LEU ARG^+^ evo2, ∆ARG LEU^++^ evo1, ∆ARG LEU^++^ evo2, and ∆ARG LEU^++^ evo3. Here the numbers indicate the origin of the ALE replicate. All CoNoS composed of a single evolved member and the corresponding non-evolved partner grew with a growth rate at least 7% higher compared to the starting CoNoS (Fig. 1C, D). The growth of ∆LEU ARG^+^ evo3 was not reproducible and this strain was thus excluded from further analysis.

In this experiment, we successfully evolved a CoNoS toward faster growth in three independent setups. The growth rate increase measured for the CoNoS with only one evolved member was lower compared to the final growth rate of the evolution experiment. This suggests that both community members carry mutations that improved growth of the community in an additive manner.

### Identification of mutations in the evolved ∆LEU ARG^+^ strain

The CoNoS ALE yielded five strains in total that presumably carried mutations beneficial for CoNoS growth. To identify and analyze these mutations in detail, we isolated genomic DNA from all five strains and sequenced it. Sequencing of the two evolved ∆LEU ARG^+^ strains yielded two mutations per strain (Table 1). Both strains carried the mutation MetC/P_brnQ_* and an additional mutation in different subunits of the Mrp1 transporter.

**Table 1:** Mutations identified by genome sequencing in evolved CoNoS strains

Strain	Position & mutation on DNA level^a^	Locus tag	Mutation	Mutation designation
∆LEU ARG^+^ evo1	SNV C281573T	cg0325	Mrp1C_G29D_	Mrp1C_G29D_
	SNV C2044002T	cg2536/P_cg2537_	MetC_S322F_/P_brnQ_*	MetC/P_brnQ_*
∆LEU ARG^+^ evo2	SNV T283546G	cg0326	Mrp1A_H335P_	Mrp1A_H335P_
	SNV C2044002T	cg2536/P_cg2537_	MetC_S322F_/P_brnQ_*	MetC/P_brnQ_*
∆ARG LEU^++^ evo1	SNV T1399043C	P_cg1504_	P_cg1504_*^1^, mutation of -35 region: ttaagg ➜ ttgagg	P_argT_*^1^
	SNV G1767718A	cg1874	Cg1874_G93D_	Cg1874_G93D_
	SNV G2712428C	cg2850	Cg2850_G30R_	Cg2850_G30R_
∆ARG LEU^++^ evo2	DEL 1399026–1401854	P_cg1504_, cg1505, cg1506	deletion of 2829 bp upstream of cg1504^b^	P_argT_*^2^
	SNV G1767718A	cg1874	Cg1874_G93D_	Cg1874_G93D_
∆ARG LEU^++^ evo3	SNV C1399000T	cg1504	cg1504*, 3rd codon GAG ➜ GAA, synonymous mutation	argT*
	SNV G1767718A	cg1874	Cg1874_G93D_	Cg1874_G93D_
	SNV G2712428C	cg2850	Cg2850_G30R_	Cg2850_G30R_

^a^Reads were mapped using GenBank accession number CP017995 (for ∆LEU ARG^+^) or BX927147 (for ∆ARG LEU^++^) as reference. Mutations are given for the plus strand. Abbreviations: deletion (DEL); single nucleotide variant (SNV)

^b^Partial deletion of the intergenic region of cg1504-cg1505, deletion of cg1505 & cg1506, partial deletion of cg1507

The mutation MetC/P_brnQ_* is located within the coding sequence of *metC* (cg2536), encoding the cystathionine β-lyase MetC [17]. This mutation was present in both strains but not in the parental strain as confirmed by sequencing of the non-evolved strain. *metC* is the first gene of a putative operon formed by *metC, brnQ* (cg2537)*,* and cg2538 [18]. *brnQ* and cg2538 are proposed to form a sub-operon with a separate transcriptional start site (TSS), which is located within the coding sequence of *metC* (Additional file 1: Fig. S2) [18]. BrnQ is a Na^+^-coupled uptake system for branched chain amino acids [19, 20]. Cg2538 encodes an uncharacterized protein annotated as putative FMN-linked alkanal monooxygenase α chain with 43.8% identity to a luciferase-like monooxygenase of *E. coli.* The mutation in *metC* caused an amino acid exchange from serine to phenylalanine at position 332. MetC S332 is moderately conserved in closely related *Corynebacterium* species and not conserved in other homologous aminotransferases of *Actinomycetales* species (Additional file 1: Fig. S3). To analyze the impact of the mutation on protein structure, we compared the best-ranked AlphaFold2 predictions for both MetC and the MetC_S322F_ variant. Superimposition of the two models yielded a C-alpha root mean square distance (C^α^ r.m.s.d.) of 0.26 Å (Additional file 1: Table S2). In fact, AlphaFold2 predicted two alternative local conformational changes in MetC_S322F_ with respect to the wildtype, compensating for the larger side chain: in the rank 1 and rank 4 models of MetC_S322F_, backbone shifting was seen, while in the other models an altered side chain torsion (χ_1_) was apparent (Additional file 1: Fig. S4A). Analysis of structural changes by Missense3D [21] revealed that S322 is largely buried in MetC, with only 2.3% relative solvent accessibility, while substitution of S322 by phenylalanine leads to enhanced surface exposure of this residue (14.2%). The hydrogen bond between the side chain hydroxyl oxygen of S322 and the carbonyl oxygen of L333 (distance 2.53 Å) is necessarily disrupted due to the S322F mutation (Additional file 1: Fig. S4A), which is accompanied by a slight shift in the polypeptide backbone around residue 333. In accordance with the annotation (see above), DALI analysis [22] of our MetC models revealed structural similarity to a putative pyridoxal-5'-phosphate (PLP)-dependent cystathionine β-lyase of *Corynebacterium diphtheriae* (PDB code 3fdb) with 55% sequence identity. Superimposition of this protein with our MetC and MetC_S322F_ models gave a C^α^ r.m.s.d. of 0.97 Å and 0.94 Å, respectively. Cystathionine β-lyases catalyze cleavage of the S-C^β^ bond in cystathionine using PLP as the cofactor, yielding homocysteine, pyruvate, and ammonia [23]. The S322F mutation occurred in helix 14 in the C-terminal domain of MetC (Additional file 1: Fig. S3). In *E. coli* MetC, the C-terminal domain is proposed to be involved in the positioning of the substrate [24]. Extending the prediction by AlphaFold2, we suspect the bulky and apolar F322 side chain to associate with a nearby hydrophobic cluster linking the C-terminal domain to an adjacent N-terminal segment. The resulting rigidification, together with the disruption of the S322–L333 hydrogen bonding, might significantly alter the conformational dynamics and thus catalytic activity of the enzyme.

Besides causing an amino acid exchange in MetC, the MetC/P_brnQ_* mutation is located 35 bp upstream of the transcriptional start site (TSS) of *brnQ* and may influence transcription of *brnQ* and the following cg2538. For *brnQ*, no specific -35 region for the major sigma factor σ^70^ was annotated [18]. The MetC/P_brnQ_* mutation increased the similarity of the -35 region of *brnQ* with the weakly conserved “ttgnca” motif by changing “tctaaa” to “tttaaa”, which may influence the transcription of the following genes.

In addition to the MetC/P_brnQ_* mutation, both strains had a mutation in the multi-subunit Na^+^/H^+^ antiporter Mrp1, which is important for environmental Na^+^ resistance and alkali tolerance [25]. Mrp1 is encoded by the gene cluster *mrp1* (cg0326-cg0321, *mrp1A, mrp1C, mrp1D, mrp1E, mrp1F, mrp1G*). The mutation in ∆LEU ARG^+^ evo1 resulted in the amino acid exchange G29D in Mrp1C, whilst the mutation in ∆LEU ARG^+^ evo2 yielded the amino acid exchange H335P in Mrp1A. The mutated positions in these proteins are highly but not fully conserved among different bacteria [25]. Thus, a mutation at these positions likely has a strong impact on Mrp1 function. Mrp1A H335 is presumably part of the ion channel and the mutation to proline at this position may alter the characteristics of the channel [25]. Mrp1C G29 is located within the first transmembrane helix of Mrp1C. The exchange of G29 to the charged amino acid l-aspartate probably has a major impact on the protein function as it may disrupt membrane insertion of the helix.

### Reconstruction of mutations in the ∆LEU ARG^+^ strain

**Fig. 2:**
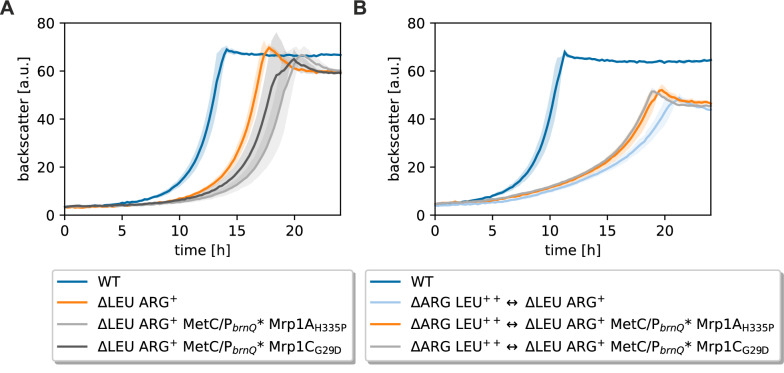
Growth performance of the reengineered *C. glutamicum* ΔLEU ARG^+^ strains. The mutations MetC/P_brnQ_* Mrp1A_H335P_ and MetC/P_brnQ_* Mrp1C_G29D_ were introduced into ΔLEU ARG^+^ and the resulting strains tested in supplemented monoculture and in a CoNoS setting. A WT monoculture was used as reference. **A** Monocultures in CGXII medium with 2% (w/v) glucose supplemented with 3 mM l-leucine (WT culture not supplemented). **B** CoNoS composed of parental and reengineered strains in comparison to the WT monoculture cultivated in in CGXII medium with 2% (w/v) glucose. Mean values and standard deviations of biological triplicates are shown as lines and shaded areas, respectively

To confirm that the identified mutations are indeed responsible for the improved growth in the CoNoS setting, all three mutations were first introduced separately into ∆LEU ARG^+^ and compared with the wild type and the parental strain ∆LEU ARG^+^ in a supplemented monoculture (Additional file 1: Fig. S5A). ΔLEU ARG+ grew slower and to a slightly lower final backscatter compared to the wild type and none of the single mutations tested improved this growth behavior (Additional file 1: Fig. S5A). This means that either more than one mutation is required to observe a positive effect or that the improved growth is only apparent in a CoNoS setting. To test this, the two double mutation strains ∆LEU ARG^+^ MetC/P_brnQ_* Mrp1A_H335P_ and ∆LEU ARG^+^ MetC/P_brnQ_* Mrp1C_G29D_ were constructed and tested under the same conditions (Fig. 2A). These two strains grew even slower than ΔLEU ARG^+^ in the supplemented monoculture, suggesting that the mutations only have a positive effect in the CoNoS setting. Thus, we combined all single and double mutation strains with the non-evolved strain ∆ARG LEU^++^ in a co-culture (Fig. 2B and Additional file 1: Fig. S5B). For the CoNoS with the single mutated strains, the growth rate slightly increased between 0.3 and 4% and the final biomass increased by approximately 4% in comparison to the parental CoNoS (Additional file 1: Fig. S5B). For the CoNoS with the double mutated strains, the growth rate and the final biomass both increased by approximately 6% in comparison to the parental CoNoS (Fig. 2B). In summary, we identified and confirmed several mutations that specifically increased growth in the CoNoS setting but not in monocultures.

### Growth with low l-leucine concentrations

Interestingly, we observed the positive effects of the MetC/P_brnQ_* mutation only in the CoNoS setting but not in monoculture, although the affected l-leucine transporter BrnQ should also be relevant during growth in l-leucine supplemented CGXII medium. Maybe the effect is only apparent when the l-leucine levels are lower than the 3 mM we used in the supplemented cultures. In a CoNoS, we expect a low amino acid concentration in the culture medium because there is a constant, moderate overproduction and concomitant consumption by the partner strain. Thus, we tested the performance of strain ∆LEU ARG^+^ MetC/P_brnQ_* Mrp1C_G29D_ in comparison to the parental strain in monoculture with l-leucine supplementation at concentrations of 100 µM–3 mM. The growth rates of both strains increased in proportion to the amount of l-leucine supplemented. However, we observed no beneficial effect of the mutations under the tested conditions (Additional file 1: Fig. S6A). It is likely that the beneficial effect is only apparent at even lower l-leucine concentrations, but there we did not see any growth in monoculture, presumably because the l-leucine concentration in the supplemented culture is not sufficient to support growth for more than a few generations.

### Influence of the MetC/P_brnQ_* mutation on transcription of ***brnQ*** and cg2538

After confirming that the evolved mutations are beneficial for CoNoS growth, we wanted to analyze their specific effect with the aim to learn more about factors limiting or promoting CoNoS growth. To test whether the MetC/P_brnQ_* mutation influences the transcription level of *brnQ* and cg2538, we measured the transcript levels of these two genes by reverse transcriptase quantitative PCR (RT-qPCR) in ∆LEU ARG^+^ and ∆LEU ARG^+^ MetC/P_brnQ_*. The *brnQ* transcript level was increased 2.1 ± 0.07-fold (1.8 ± 0.28-fold in an independent experiment) in ∆LEU ARG^+^ MetC/P_brnQ_* compared to the control strain. In contrast, transcription of cg2538 was almost unchanged (1.13 ± 0.04-fold). Thus, it seems that *brnQ* and cg2538 are transcribed independently, which has also been observed in other studies [26, 27], and there might be a separate TSS in front of cg2538. Thus, Cg2538 is most likely not responsible for the effect of the MetC/P_brnQ_* mutation. In summary, the MetC/P_brnQ_* mutation increased *brnQ* transcription and presumably the BrnQ content of the cell. As BrnQ activity is regulated based on de novo synthesis [28], we assume that the mutation caused elevated l-leucine uptake of the cell, which then increased growth in the CoNoS setting.

### Effects of mutations in Mrp1 during growth with elevated NaCl concentrations

**Fig. 3:**
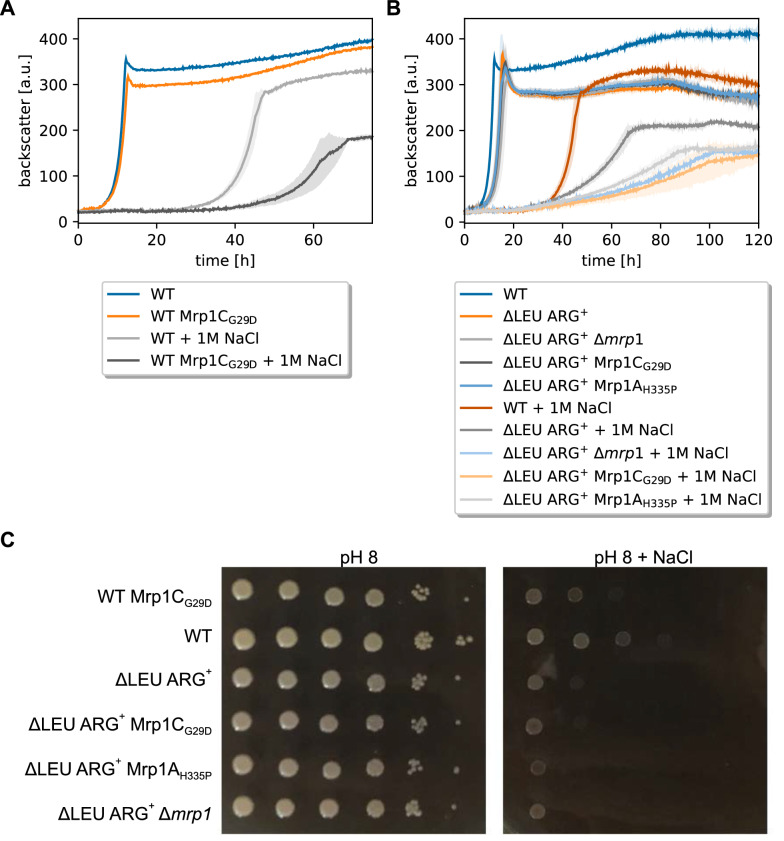
Growth performance of Mrp1 mutant strains in supplemented monocultures under NaCl stress conditions. **A** Monocultures of WT and WT Mrp1C_G29D_ cultivated with 2% (w/v) glucose. **B** Monocultures of mutated ∆LEU ARG^+^ strains in comparison to WT, ∆LEU ARG^+^. For (**A**, **B**) cultures were performed in biological triplicates in CGXII medium with 2% (w/v) glucose, supplemented with 3 mM l-leucine for auxotrophic strains. Mean values and standard deviations are shown as lines and shaded areas, respectively. **C** Tenfold serial dilutions of different *C. glutamicum* Mrp1 mutant strain cultures. Cells were precultivated in test tubes at 30 °C 180 rpm for 8 h in BHI medium. Afterwards, second precultures were prepared in CGXII with 2% (w/v) glucose and 3 mM l-leucine for the auxotrophic strains and cultivated over night at 30 °C 180 rpm. Dilutions were prepared in PBS starting with an OD_600_ of 1 and spotted onto CGXII-agar plates (pH 8.0, 2% (w/v) glucose, 3 mM l-leucine) with different NaCl concentrations (0 M and 0.6 M) and incubated at 30 °C for 48 h, as described elsewhere [25]

Besides the MetC/P_brnQ_* mutation, the evolved strains carried mutations in different subunits of Mrp1. To test the effect of Mrp1C_G29D_ on sodium resistance and alkali tolerance without side effects of other mutations, we reconstructed this mutation in the wild type background resulting in strain WT Mrp1C_G29D_. We analyzed the sodium resistance of this strain in comparison to the wild type in a growth experiment with liquid CGXII medium containing 2% (w/v) glucose as carbon source and either no or 1 M NaCl (Fig. 3A). Without NaCl, growth rate and final backscatter of WT Mrp1C_G29D_ were only slightly decreased compared to the wild type. With 1 M NaCl, the growth rate and the final backscatter were more than 40% decreased for WT Mrp1C_G29D_ in comparison to the wild type. When the two strains were cultivated on CGXII agar plates, both strains grew similarly in the absence of NaCl, while WT Mrp1C_G29D_ grew much worse than the wild type in the presence of 1 M NaCl (Fig. 3C and Additional file 1: Fig. S7). The Mrp1 transporter is especially relevant under alkaline conditions, thus the plate assays were performed both at pH 7 and pH 8, but there was no obvious difference between the two conditions. The results suggested that the G29D mutation strongly impairs, if not completely blocks, the function of Mrp1C.

To test the effects of the Mrp1C_G29D_ and Mrp1A_H335P_ mutations in the auxotrophic strain background, we constructed strains ∆LEU ARG^+^ Mrp1C_G29D_ and ∆LEU ARG^+^ Mrp1A_H335P_ as well as the deletion mutant ∆LEU ARG^+^ Δ*mrp1*, lacking the whole *mrp1* gene cluster. These strains were compared to the parental strain and to the wild type in liquid medium and on agar plates regarding sodium resistance and alkali tolerance. In liquid medium without NaCl addition, all three strains grew like the parental strain ∆LEU ARG^+^, which is slightly slower than the wild type (Fig. 3B). In liquid medium with 1 M NaCl, ∆LEU ARG^+^ grew much slower and to a lower final backscatter than the wild type, which means that this strain had already lost some of its ability to cope with NaCl stress (Fig. 3B). Deletion of *mrp1*, as well as mutations Mrp1C_G29D_ and Mrp1A_H335P_, impaired growth further, which strongly suggested that the mutations led to a loss of function. On solid medium, strain ∆LEU ARG^+^ already grew much worse than the wild type (Fig. 3C and Additional file 1: Fig. S7). Upon mutation or deletion of *mrp1*, there was no further impact on growth visible, possibly because the effect was masked by the already reduced growth of ∆LEU ARG^+^. In summary, we observed that both mutations Mrp1C_G29D_ and Mrp1A_H335_ had a similar negative effect in the presence of elevated NaCl concentrations like the entire deletion of *mrp1*. However, we do not know yet why a loss of Mrp1 function is beneficial for CoNoS growth.

### Identification of mutations in the evolved ∆ARG LEU^++^ strain

Sequencing of the ∆ARG LEU^++^ strains isolated in the ALE revealed two or three mutations per strain (Table 1). The mutations occurred in two proteins of unknown function (Cg1874 and Cg2850) and close to the TSS/TLS (translational start site) of cg1504-1502 (named *argTUV* from here onwards), which encode a putative ABC-type amino acid transport system for polar amino acids. Interestingly, identical mutations for cg1874 as well as cg2850 occurred in more than one evolution setup. Sequencing of the relevant regions of the non-evolved strains confirmed that these mutations must have appeared independently during evolution.

Cg1874 and Cg2850 are uncharacterized proteins with homologs in closely related species and other *Actinomycetota* genera such as *Arthrobacter* or *Rhodococcus*. To get an idea about their function and the effects of the mutations we performed AlphaFold2 prediction. For Cg2850, superimposition of the top-ranked predicted WT and variant structures resulted in a C^α^ r.m.s.d. of 0.21 Å (residues 1–27 are predicted to be disordered and were excluded from the comparison) (Additional file 1: Table S2). DALI analysis of the Cg2850 models showed high structural similarity (C^α^ r.m.s.d. of 1.89 Å and 1.85 Å for WT and mutant, respectively) and 48% sequence identity with Rv0813c (PDB code 2fwv), a fatty acid binding protein-like protein of *Mycobacterium tuberculosis* [29]. Rv0813c belongs to the calycin superfamily implicated in the transport and storage of small and often hydrophobic molecules. Therefore, it seems conceivable that Cg2850 may participate in shuttling of amino acids or their metabolites, e.g. to/from membrane transporters. In the predicted Cg2850 structure, as observed in Rv0813c, residues 80–229 appear to form an anti-parallel β-barrel which could provide a cavity for non-covalent interactions (binding of ligands), while residues 28–79 constitute a partly helical N-terminal segment that mostly lines the circumference of the barrel on one end and becomes increasingly dynamic toward the N-terminus (Additional file 1: Fig. S4B). This region is relatively conserved between Rv0813c and its orthologs in other *Mycobacteria* and *Corynebacteria* and is proposed to have an important role in mediating protein–protein interactions [29]. The site of mutation (G30), in particular, is conserved across many *Corynebacteria*, *M. tuberculosis, Mycolicibacterium smegmatis,* and *Rhodococcus rhodochrous* (Additional file 1: Fig. S9). Interestingly, the G30R exchange, at the beginning of the N-terminal α-helix, generates a potential salt bridge with E169 on the β-barrel. While the respective atoms are 3.67 Å apart in the model, a favorable hydrogen bonding distance is easily achievable by choosing different rotamers. Concomitantly, a slight decrease in the internal cavity volume (by 43 Å^3^) was predicted by Missense3D in the Cg2850_G30R_ variant. Formation of the salt bridge at the base of the presumed ligand-binding cavity of Cg2850_G30R_ might therefore affect the overall activity of the protein by restraining the dynamics of the N-terminus. Interestingly, this mutation is about 130 bp upstream of the TSS of cg2849, a putative kinase related to diacylglycerol kinase, and may influence its transcription.

For Cg1874, DALI suggested structural similarity of our AlphaFold2 models to a variety of proteins lacking obvious mutual relationship. These hits are most likely spurious and related to a shared helical bundle topology. While the PDB does not seem to contain entries related to Cg1874, sequence-based analysis using InterPro [30] revealed the presence of a DUF3817 domain in its N-terminal half. This domain of unknown function contains two predicted transmembrane helices and is occasionally found as part of larger membrane proteins such as transporters of the major facilitator superfamily, prokaryotic members of which are involved in nutrient uptake. Given that Cg1874 is a standalone protein, we speculate that it may function as a subunit or modulator of an amino acid transporter in *Corynebacteria*. It has 48% identity to a DUF3817 domain-containing protein from *Arthrobacter gandavensis*, 38% to a membrane protein of *R. rhodochrous* and 36% identity to a membrane protein of *Mycobacterium gallinarium* (Additional file 1: Fig. S8). Despite the mutation from glycine to a charged residue (G93D) and even though the mean pLDDT (predicted local distance difference test) for the mutated variant was higher than for the WT (Additional file 1: Table S2), no prominent structural changes were observed (Additional file 1: Fig. S4C). In fact, the C^α^ r.m.s.d. between WT and mutated structures amounts to only 0.13 Å for this protein. A multiple sequence alignment (MSA) with the sequences of Cg1874 homologs showed a full conservation of G93 (Additional file 1: Fig. S8). In an additional alignment of 980 sequences performed by PredictProtein [31], 20 protein sequences had a D in the position corresponding to G93, which may explain why the G93D exchange did not cause notable disturbance in the predicted structure. We also note that the site of mutation is about 200 bp upstream of the TSS of cg1873, a putative acyl-CoA thioesterase II protein, the expression of which may be affected.

The mutations related to *argTUV* were all close to the TSS/TLS in front of *argT* [18] (Additional file 1: Fig. S10). *argT* is transcribed leaderless, which means that TSS and TLS are identical. The proteins encoded by *argTUV* show high similarity to ABC-type transport systems for polar amino acids. Here, ArgT is a homolog to the secreted component with 46% identity to the glutamine-binding periplasmic protein of *Gordonia paraffinivorans* and 46% identity to the substrate-binding protein of the polar amino acid transport system of *R. triatomae.* It was proposed that the three genes *argTUV* encode an uptake system for polar amino acids such as l-arginine, l-citrulline or l-ornithine, but experimental evidence is missing so far [32, 33].

For mutation P_argT_*^1^, the single nucleotide variant (SNV) 35 bp upstream of the TSS changed the sequence in such a way (ttaagg ➜ ttgagg, Additional file 1: Fig. S10) that the −35 region became more similar to the reported −35 recognition sequence hexamer “ttgnca” which could lead to an increased transcription of *argTUV* [18]. In the strain with mutation P_argT_*^2^, 2829 bp were deleted upstream of the *argT* TSS. Besides deletion of cg1505 and cg1506 there was also a partial deletion of cg1507 and a partial deletion of the intergenic region between cg1504-cg1505 which led to an altered −35 region that does not really fit to the consensus motif ttgnca (Additional file 1: Fig. S10). Thus, we cannot expect a positive effect on promoter activity here. However, transcription of *argTUV* could be influenced by promoters that are further upstream that read through to *argTUV* due to the deleted region and potentially missing terminators. These promoters are, for example, of cg1507 (phage-type integrase), cg1513 (transposase), cg4005 (putative secreted protein) or of cg1514 (secreted protein). In the strain with mutation argT*, there is a synonymous mutation of the third ArgT codon from GAG to GAA. Both codons are frequently used in *C. glutamicum* (43.8% and 56.2%, respectively) [34], so we cannot expect a major effect on ArgT translation due to altered codon usage here. As stated above, *argT* is a leaderless transcript and until now it is not fully understood how translation initiation and regulation works for these transcripts [35]. It is known, however, that downstream elements such as CA multimers can improve translation speed in *E.* *coli* [36], presumably through the provision of a lack of structure, since secondary mRNA structures immediately downstream of the AUG were shown to influence translation efficiency of leaderless mRNA [37]. This structural effect might also account to the *argTUV* mRNA with the G ➜ A mutation, resulting in an increased l-arginine importer level.

### Reconstruction of mutations in the ∆ARG LEU^++^ strain

**Fig. 4:**
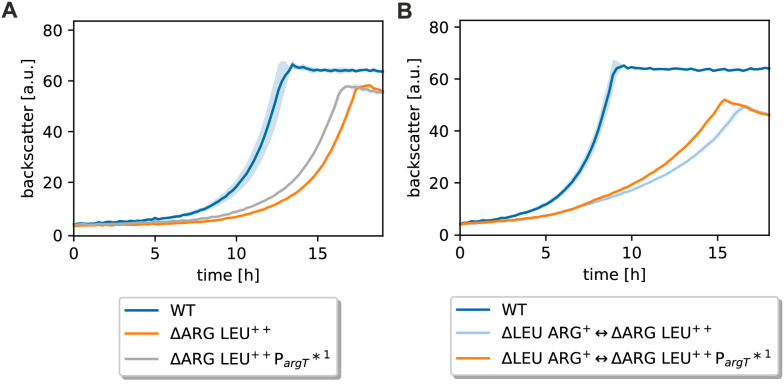
Growth performance of ΔARG LEU^++^ strains with reengineered mutation P_argT_*^1^ in supplemented monocultures and in the CoNoS setting. **A** Monoculture of mutated strain in comparison to the WT and ∆ARG LEU^++^ cultivated in CGXII medium with 2% (w/v) glucose and 3 mM l-arginine. **B** Comparison of WT monoculture, CoNoS consisting of non-evolved strains and CoNoS containing one mutated strain in CGXII medium with 2% (w/v) glucose. Mean values and standard deviations of biological triplicates are shown as lines and shaded areas, respectively

To test which of the identified mutations are responsible for improved growth in the CoNoS setting, we first reconstructed the mutation of ∆ARG LEU^++^ evo1 in ∆ARG LEU^++^ yielding ∆ARG LEU^++^ P_argT_*^1^. This strain was compared to the parental strain both in supplemented monoculture and in the CoNoS setting. In monoculture, the mutated strain appeared to start growing a bit earlier, but the growth rate was similar (Fig. 4A). In contrast, the growth rate of the CoNoS containing the strain ∆ARG LEU^++^ P_argT_*^1^ was about 13% higher compared to the CoNoS containing the non-mutated parental strain (Fig. 4B). In summary, we did not see any positive effect of the mutation P_argT_*^1^ on growth in monoculture, but we confirmed that it had a positive effect in the CoNoS setting.

To test whether the synonymous mutation of the third *argT* codon has a similar effect, we reconstructed this mutation in ∆ARG LEU^++^ yielding ∆ARG LEU^++^ argT*. In monoculture, this strain grew essentially like ∆ARG LEU^++^ P_argT_*^1^ (Additional file 1: Fig. S11A), which grew significantly better than the parental strain ∆ARG LEU^++^ (Fig. 4A). A CoNoS containing ∆ARG LEU^++^ argT* grew slightly slower than the CoNoS containing ∆ARG LEU^++^ P_argT_*^1^ (Additional file 1: Fig. S11B), but presumably better than the parental CoNoS ∆LEU ARG^+^ ⇔ ∆ARG LEU^++^ (Fig. 4B). This matches the results from Fig. 1C that the CoNoS including strain ∆ARG LEU^++^ evo3 did not grow as good as the CoNoS with the two other evolved strains. As the beneficial effect of the mutation P_argT_*^1^ appeared stronger compared to the effect of argT^*^, we decided to only study the first one further.

To investigate the role of Cg1874_G93D_ and Cg2850_G30R_, these mutations were additionally introduced into ΔARG LEU ^++^ P_argT_*^1^*.* The newly constructed strains did not show growth differences compared to their parental strain both in monoculture and in the CoNoS setting (Additional file 1: Fig. S12). Thus, we assume that the two mutations Cg1874_G93D_ and Cg2850_G30R_ play a less important role than the *argT* related mutations for CoNoS growth.

### Effects of the *argTUV* related mutations

**Fig. 5:**
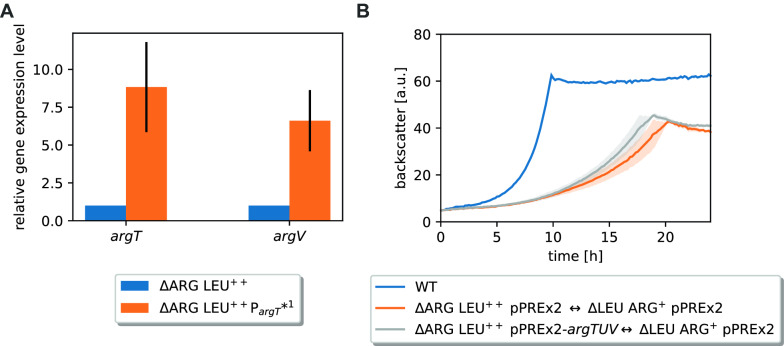
Analysis of *argTUV* expression levels and their effects in a CoNoS setting. **A** Relative gene expression levels of *argT* and *argV* analyzed via RT-qPCR. **B** Growth performance of CoNoS with strains harboring either empty pPREx2 or pPREx2-*argTUV*. WT monoculture without amino acid supplementation is shown as reference. All strains were pre-cultivated in CGXII medium with 2% (w/v) glucose and 3 mM of the required amino acid. Mean values and standard deviations of three biological replicates are shown as lines and shaded areas, respectively

The mutations P_argT_*^1^ and argT* both improved the growth in the CoNoS setting. P_argT_*^1^ had a slightly stronger effect than argT*, thus we decided to study this mutation further. P_argT_*^1^ changed the −35 sequence in such a way that the –35 region became more similar to the reported −35 recognition sequence. We wanted to know whether this mutation has an impact on transcription and analyzed the expression levels of *argT* and *argV* in ∆ARG LEU^++^ and ∆ARG LEU^++^ P_argT_*^1^ by RT-qPCR. The expression level of *argT* was increased by 8.83 ± 2.97 fold and expression of *argV* was increased by 6.61 ± 2.02 fold in ΔLEU ARG^+^ P_argT_*^1^ compared to the parental strain (Fig. 5A). As expected, the mutation in the −35 region led to an increased transcription of *argTUV*, which is presumably responsible for the improved growth of the strain in the CoNoS setting. To further prove this, we tested whether plasmid-based overexpression of *argTUV* has a similar effect. We compared growth of ΔARG LEU^++^ pPREx2-*argTUV* to ∆ARG LEU^++^ pPREx2 in a CoNoS with ΔLEU ARG^+^ pPREx2 in CGXII medium with 2% (w/v) glucose. Even without isopropyl β-d-1-thiogalactopyranoside (IPTG) addition, the CoNoS including strain ARG LEU^++^ pPREx2-*argTUV* had an 18% increased growth rate compared to the control CoNoS containing just the empty plasmid (Fig. 5B). Induction of *argTUV* transcription with 50 or 250 µM IPTG did not have a further positive effect (data not shown).

In the strain with mutation P_argT_*^2^, a larger region upstream of *argT* is deleted including the two genes cg1505 (putative secreted protein) and cg1506 (putative membrane protein). To test whether the deletion of these two genes has an effect on growth, we constructed strain ∆ARG LEU^++^ ∆cg1505-cg1506. Deletion of those two genes did not affect the growth rate of a supplemented monoculture or of a CoNoS (Additional file 1: Fig. S11C, D).

Subsequently, we tested whether a higher *argTUV* expression enables ∆ARG LEU^++^ P_argT_*^1^ to grow faster than ∆ARG LEU^++^ at lower levels of l-arginine in the growth medium. The growth rates of both strains increased in proportion to the amount of l-arginine supplemented. However, no beneficial effect of the mutation was observed (Additional file 1: Fig. S6B).

### Characterization of ArgTUV as l-arginine importer

**Fig. 6:**
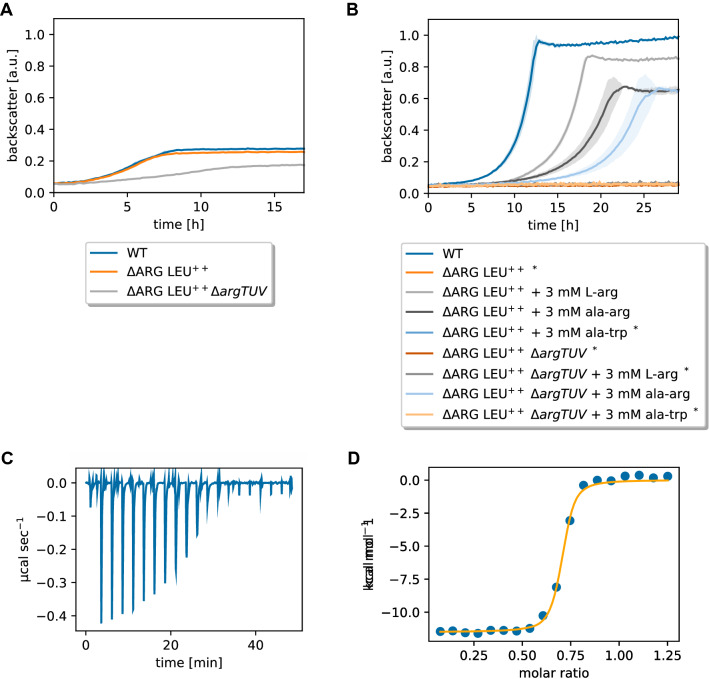
Characterization of ArgTUV as l-arginine importer. **A** Comparison of ∆ARG LEU^++^ Δ*argTUV* to the parental strain and the WT during growth in BHI medium. All strains were precultivated in BHI medium. **B** Comparison of ∆ARG LEU^++^ Δ*argTUV* to the parental strain and the WT in CGXII with 2% (w/v) glucose supplemented with l-arginine or dipeptides as indicated. Strains marked with ^*^ did not grow. The WT monoculture without amino acid supplementation is shown as reference. ∆ARG LEU^++^ Δ*argTUV* was pre-cultivated in BHI, all other strains were pre-cultivated in CGXII medium with 2% (w/v) glucose and 3 mM of the required amino acid. **A**, **B** Backscatter data were normalized by the maximum value recorded for the WT monoculture. Mean values and standard deviations of three biological replicates are shown as lines and shaded areas, respectively. **C** Raw data of an ITC experiment with 200 µM l-arginine and 30 µM His_10_-ArgT in 40 mM HEPES–NaOH buffer, pH 7.4, with 100 mM NaCl. **D** Corresponding binding isotherm created by plotting the integrated heat peaks against the molar ratio

To test whether ArgTUV indeed plays a role in l-arginine uptake, we deleted the respective operon in the l-arginine auxotrophic strain ∆ARG LEU^++^. In BHI medium, the resulting strain ∆ARG LEU^++^ Δ*argTUV* grew slower and to a lower final backscatter compared to the parental strain and the WT (Fig. 6A). When the same strains were cultivated in CGXII medium with 2% (w/v) glucose and 3 mM l-arginine, ∆ARG LEU^++^ grew slightly slower and to a lower final backscatter compared to the wild type (Fig. 6B). Strain ∆ARG LEU^++^ Δ*argTUV* did not grow (Fig. 6B), which suggested that ArgTUV is indeed an l-arginine importer and obviously the only one active in *C. glutamicum* under the tested conditions. This was further confirmed by the fact that ∆ARG LEU^++^ Δ*argTUV* was able to grow when the medium was supplemented with an ala-arg dipeptide, but not with an ala-trp dipeptide (Fig. 6B).

Based on significant sequence homology to other substrate binding proteins such as ArtJ of *Geobacillus stearothermophilus* and hypothetical ancient precursors binding l-arginine as well as further amino acids [38, 39], ArgT was assumed to be the l-arginine binding component of the ABC-transporter ArgTUV. To characterize its ligand binding properties, an ArgT variant with cleavable His-tag and lacking the signal peptide (His_10_-ArgT) was overproduced in *E.* *coli* BL21(DE3) pET-TEV-*argT* and purified by Ni–NTA affinity chromatography and size exclusion chromatography (SEC). The purified protein (Additional file 1: Fig. S13) eluted in two peaks from the SEC column (corresponding to monomer and trimer/tetramer) and was used for ligand interaction studies by isothermal titration calorimetry (ITC). Of the tested ligands, only l-arginine and l-citrulline, but not l-histidine, l-glutamate, l-glutamine, l-lysine or l-cysteine were bound by ArgT (Additional file 1: Table S3). Figure 6C shows a representative ITC experiment for His_10_-ArgT binding l-arginine as a ligand. A representative ITC experiment with l-citrulline is shown in Additional file 1: Fig. S14. From four experiments of two independent His_10_-ArgT purifications, a mean equilibrium dissociation constant (*K*_D_) of 29.5 ± 4.8 nM was obtained for l-arginine. For l-citrulline, a ten times higher mean *K*_D_ of 432 nM (one measurement of one purification) was determined. For both ligands, exothermic binding was measured, for l-arginine with a mean enthalpy change of -12.2 ± 1.1 kcal/mol. These results confirmed that ArgTUV is a high affinity uptake system for l-arginine and may also transport l-citrulline.

### Evolution-guided metabolic engineering of CoNoS ∆LEU ARG^+^ ↔ ∆ARG LEU^++^

**Fig. 7:**
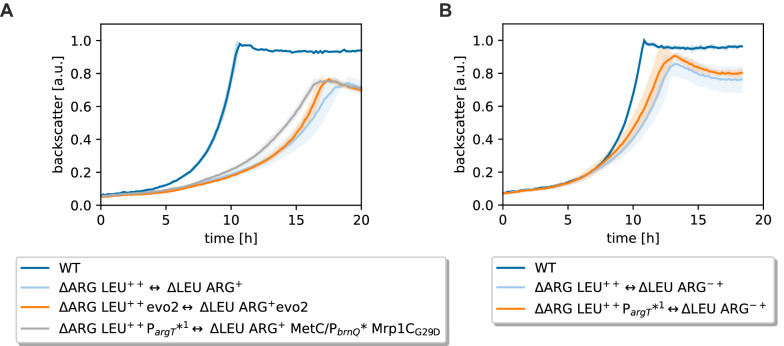
Growth performance of reengineered CoNoS and effect of beneficial mutation on an a priori better growing CoNoS. **A** Reengineered CoNoS ∆ARG LEU^++^ P_argT_*^1^ with reengineered ΔLEU ARG^+^ MetC/P_brnQ_^*^ Mrp1C_G29D_ in comparison to the parental CoNoS, to the evolved CoNoS and to a WT monoculture. **B** Rationally optimized CoNoS with the additional P_argT_*^1^ mutation in comparison to the parental CoNoS and WT monoculture. Mean values and standard deviations of biological triplicates in CGXII medium are shown as lines and shaded areas, respectively

In the previous sections, we described that single reconstructed strains improved the growth rate of a CoNoS containing either ∆LEU ARG^+^ MetC/P_brnQ_* Mrp1C_G29D_ or the ∆ARG LEU^++^ P_argT_*^1^ by 6% and 13%, respectively (Figs. 2B, 4B). Hence, we wanted know whether the positive effects are additive and combined these two reconstructed strains in a new CoNoS. This culture reached a growth rate of 0.27 h^−1^, corresponding to a 21% increase compared to the parental CoNoS (Fig. 7A). Furthermore, the final backscatter value increased by 7% (Fig. 7A). This confirmed that the mutations in both strains increased growth rate of the CoNoS in an additive manner (summarized in Additional file 1: Fig. S15). Finally, the CoNoS with the two reconstructed strains grew with a similar median growth rate like the CoNoS consisting of two evolved strains (Fig. 7A), which suggested that we found all relevant major mutations.

For the ALE described in this paper, we chose a CoNoS with limited growth leaving much room for improvement. After confirming the beneficial effect of the single identified mutations on CoNoS growth, we wanted to know whether these mutations further improve a CoNoS so far only optimized by rational engineering. In our previous paper, we described the CoNoS C1*∆LEU ARG^++^ ↔ WT∆ARG LEU^++^ as the best CoNoS available to date [9]. In the meantime, we also tested a CoNoS with strains WT∆LEU ARG−+ (ΔLEU ARG^−+^) ↔ WT∆ARG LEU^++^ where ∆LEU ARG^−+^ only carries the mutations ArgB_A26V M31V_ but not the deletion of *argR* (Please note the superscript “− + ” to distinguish this strain from the one that only carries the Δ*argR* mutation). This CoNoS performed equally well as the best CoNoS described in [9] and was chosen to test the effect of an additional mutation that we discovered here during this ALE.

We introduced the mutation P_argT_*^1^ into WT∆ARG EU^++^ and co-cultivated this strain with WT∆LEU ARG^−+^. Here, we indeed observed an increase in growth rate of 7% and an increase in final backscatter of 6% on comparison to the parental CoNoS (Fig. 7B). This demonstrated nicely that the ALE can reveal beneficial mutations for CoNoS growth that are not immediately obvious and that can help to further optimize rationally designed CoNoS.

### Effects of *argTUV* deletion on l-arginine production in supplemented monoculture

**Fig. 8:**
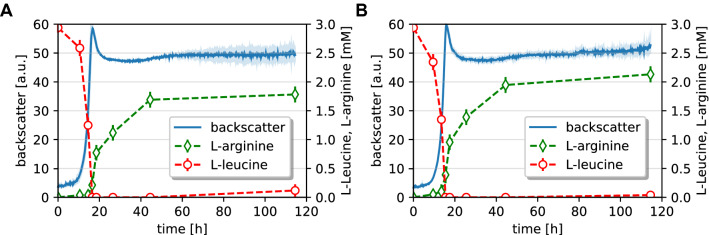
Characterization of l-arginine production in monocultures of **A** ∆LEU ARG^++^ and **B** ∆LEU ARG^++^ ∆*argTUV* in CGXII with 2% (w/v) d-glucose and 3 mM l-leucine. After precultivation in CGXII defined medium with 111 mM d-glucose and 3 mM l-leucine, the cell suspension of one culture was used to inoculate eight wells of a BioLector Flowerplate. Upon sampling, a full well was harvested for each replicate at a certain time point. l-arginine and l-leucine were quantified in cell-free supernatants via HPLC and mean values are represented including the corresponding standard deviation. Mean values and standard deviations of biological triplicates are shown as lines and shaded areas, respectively

In some cases, the deletion of specific amino acid importers is beneficial for the production of the transported amino acid. To test whether this is also the case for ArgTUV, the transporter was deleted in ∆LEU ARG^++^ that produces moderate amounts of l-arginine in monoculture. We compared ∆LEU ARG^++^ Δ*argTUV* to the parental strain regarding growth and l-arginine production in a monoculture. The growth of both strains was similar (Fig. 8). Determination of the l-arginine titer in the supernatants by HPLC revealed a significant increase in l-arginine by 24% after around 26 h, and by 20% after 115 h (Fig. 8). Notably, the titer difference was particularly clear in the early cultivation stage (14 h), where the l-arginine accumulation was more than 150% higher in ∆LEU ARG^++^ Δ*argTUV*. However, most of the l-arginine was produced in the late exponential and in the stationary phase.

In summary, we showed that the deletion of *argTUV* had a positive effect on external l-arginine accumulation under the tested conditions. This emphasizes the potential to identify beneficial mutations during ALE of a CoNoS which also have a positive effect on metabolite production with monocultures.

## Discussion

Native microbial communities have usually evolved over thousands of years toward an extremely efficient use of the available resources, thereby heavily relying on cooperation and cross-feeding among the community members [1, 3, 5]. Recent advances in evolving synthetic co-cultures of strains of the same species [6, 40] or mixed species communities [41, 42] underlined the potential of increasing product cross-feeding, which could be of high biotechnological value. In this work, we successfully evolved a synthetic community composed of amino acid auxotrophic strains [9], identified the relevant mutations and used these to increase l-arginine production also in monoculture.

Automated ALE using repetitive batch cultures, which has so far almost exclusively been demonstrated for monocultures [15, 43], proved to be easily applicable for CoNoS to select for faster-growing communities. During the ALE, both the ΔARG and the ΔLEU strains accumulated mutations beneficial for community growth, which was also observed for synthetic co-cultures consisting of l-leucine and l-lysine auxotrophic *E. coli* strains [44]. Several mutations we found increased growth in a co-culture setting, but did not result in better growth of a monoculture supplemented with the required amino acid. This is also in agreement with the *E. coli* approaches, where single evolved community members showed decreased growth in supplemented monocultures [44].

Let’s have a closer look on the mutations we found in the evolved CoNoS. These were i) a mutation in the cystathionine β-lyase MetC, ii) mutations in or upstream of amino acid uptake systems, iii) mutations in the multi-subunit Na^+^/H^+^ antiporter Mrp1, and iv) mutations in uncharacterized genes. Based on our MSA and AlphaFold2 data, we proposed the MetC S322F mutation to alter the dynamics of the C-terminal domain, thus modulating substrate binding and/or catalysis. In the context of the ∆LEU ARG^+^ strain, the resulting changes to the steady-state levels of homocysteine, cystathionine, and methionine may indirectly influence either l-leucine utilization or l-arginine export. Conflicting results regarding the applicability of AlphaFold2 for predicting the impact of single point mutations in protein structures have been reported [45–48]. In the case of MetC and Cg2850, despite the high similarity of the structures predicted for the WT and the mutated version, small structural changes can be detected in the vicinity of the mutation site. This was not observed for Cg1874. Notably, even if an amino acid exchange does not significantly alter the mean structure, it may nevertheless affect other properties such as protein dynamics, stability, enzymatic activity, or protein–protein and protein–ligand interactions, the investigation of which is beyond the scope of this study.

In our CoNoS with auxotrophic strains, efficient amino acid export and uptake appears to be one of the key factors for community growth. In total, we found four different mutations that presumably increased the amount of available transporters in the cell (Table 1). In this context, we identified and characterized ArgTUV as an l-arginine and l-citrulline importer. Despite ArgT showing significant homology to other secreted substrate-binding proteins such as ArtJ of *G. stearothermophilus* and hypothetical ancient precursors binding also l-histidine, l-lysine, l-cysteine or l-glutamine [38, 39], ArgT bound exclusively to l-arginine and its molecular precursor l-citrulline. When synthetic communities of two *E. coli* strains auxotrophic for histidine or one other metabolite were evolved, several mutations appeared in promoter and regulatory regions that increased e.g. l-histidine and 2-oxoglutarate uptake [40]. Only very few mutations were found in the coding region of transporters, which might alter transporter activity or codon usage or translation by influencing mRNA structure [40]. In a further study, two *E. coli* strains auxotrophic for either l-tryptophan or l-tyrosine were evolved together and the resulting strains produced more of the amino acid required by the partner strain [6]. The evolved strains were not sequenced, thus it is unknown whether also other factors, such as amino acid import, was affected [6]. In another study with a co-culture consisting of two *E. coli* strains auxotrophic for either l-tryptophan or l-tyrosine, mutations were identified in a porin and in the global transcriptional regulator Lrp [49]. The evolution of a lactic acid bacterium, which is naturally auxotrophic for amino acids, together with a *Saccharomyces* *cerevisiae,* auxotrophic for riboflavin or folate, also revealed several mutations that regulate transcription or are associated with amino acid uptake [41]. Interestingly, most of the mutations influenced transcription or translation of the transporter protein, and only a few the activity of the protein itself, and mutations were almost exclusively associated with uptake systems but not with exporters.

The Mrp1 mutations resulted in severe impairments of the function of the multi-subunit Na^+^/H^+^ antiporter Mrp1, analogous to gene deletion or other mutations identified in Mrp1 subunits before [25, 50]. The fact that mutations of Mrp1 and the MetC/P_brnQ_* mutation evolved together twice in independent experiments suggested a functional link between these two proteins. In most organisms, the l-leucine-import via BrnQ depends on the proton motive force (reviewed in [51]), coupling l-leucine and Na^+^-symport across an energy gradient [52]. Mrp1 is the main Na^+^/H^+^ antiporter in *C. glutamicum* and required to establish the gradient for Na^+^-coupled uptake [20, 50]. A defect in Mrp1 presumably leads to a decreased Na^+^ gradient and thus a reduced l-leucine import. Therefore, it is not obvious how the Mrp1 mutations are beneficial for the ΔLEU strain.

The fact that the mutations in the uncharacterized proteins Cg1874 and Cg2850 evolved several times independently from each other is a strong hint that they may be somehow beneficial for CoNoS growth. Their specific role is still unclear, as their reconstruction had no obvious effect in monoculture and in a CoNoS setting (Additional file 1: Fig. S12). However, the reconstructed strains were only tested in a CoNoS with the parental ΔLEU ARG^+^ strain, so maybe the beneficial effect is only apparent with a partner carrying mutations MetC_S322F_/P_brnQ_* and/or mutations in Mrp1.

In summary, these results suggested that the metabolite uptake is often the major bottleneck under the very low metabolite concentrations in a CoNoS as observed before [9]. The identified mutations support the view that transport may be mostly limited by the availability of transporter proteins, because all mutations presumably led to an increased transporter availability. Metabolite production and export appears to be less critical in our setup because we did not find any mutation obviously related to these processes. Nevertheless, rationally increased amino acid production also increased the community growth, suggesting that sufficient amino acid production is still one major bottleneck, leaving room for improvement. For the rational design of synthetic communities, this means that one should concentrate both on metabolite production and on metabolite import to obtain optimal community growths.

At the end of this study, we would like to discuss what kind of mutations we expected to find by evolution of a CoNoS and how this differs from the ALE of monocultures. When selecting for faster growing strains or cultures, the selection pressure is highest on the bottleneck that is limiting growth most strongly. In our case, this was most likely amino acid import, because we found mutations in promoters leading to an increase in l-leucine and l-arginine import. Elevated uptake can not only result from promoter mutations that promote RNA polymerase binding upstream of the importer gene, but also be caused by mutations of regulators, mutations of the transporter start codon to a more favorable one, mutation of the RBS, mutation of the transporter itself increasing binding affinity or transport speed, mutations that lead to more favorable codons and several other mechanisms reviewed elsewhere [53, 54]. Thus, there are numerous potential targets which can mutate to increase import. If export is the limiting factor, the transporter and the corresponding regulatory mechanisms can mutate in a similar way. If transport is no longer limiting, we would also expect mutations in the amino acid biosynthesis pathways themselves. Here, again, regulatory processes can be affected, or the biosynthetic enzymes mutate to release e.g. feedback inhibition or increase reaction speed. Thus, to find mutations in the biosynthetic pathways using ALE, it is necessary to generate a CoNoS that is no longer limited in amino acid import and export.

## Conclusions

Even after decades of research, the genome annotations of *C. glutamicum* and other biotechnologically relevant production organisms still contain many uncharacterized segments, harboring potential for not only increasing metabolic understanding but also for increasing metabolite production. The co-culture evolution-guided metabolic engineering approach presented in this study represents one additional tool for exploiting this potential through putting higher selective pressure on communities to grow faster comparable to monoculture approaches. This enabled the identification of amino acid transport systems not identifiable with other evolution approaches so far. Especially the deletion of the identified *argTUV* in existing high-yield l-arginine-producers [55] could therefore be worthwhile. Co-culture evolution-guided metabolic engineering could also easily be extended not only to other CoNoS published before [9] but also to a number of different metabolite cross-feeding pairings, enabling the identification of more transport systems. Additionally, further rounds of evolution with already import-optimized strains with lower levels of production could result in mutations occurring also in metabolic pathway enzymes, since also other co-culture pairings suggested that increasing production via community evolution is possible [6]. Employing the new best CoNoS for future work will enable further progress in improving small molecule production with highly efficient microbial communities [3].

## Methods

### Bacterial strains and growth conditions

The microbial strains used in this study are listed in Table 2. *C. glutamicum* strains are based either on the wild type *C. glutamicum* ATCC13032 or on its genome reduced variant C1* [56]. Microbial cultivations of *E. coli* and *C. glutamicum* were performed as described [9]. *C.* *glutamicum* was cultivated at 30 °C in brain heart infusion (BHI) medium (Difco Laboratories, Detroit, USA) or defined CGXII medium [57], notably with 0.03 g L^−1^ of protocatechuic acid (PCA). *E. coli* was cultivated at 37 °C in lysogeny broth (LB) [58] or on LB agar plates, with addition of 50 µg mL^−1^ kanamycin when plasmid bearing strains were used. For analyzing sodium sensitivity, NaCl was added to a concentration of 1 M to CGXII medium after autoclaving it separately [50]. CGXII agar plates were prepared similarly to liquid medium with additional 9 g L^−1^ agar. NaCl was added to a concentration of 0.6 M if appropriate [25].

**Table 2:** Bacterial strains used in this study

Strain (abbreviation)	Characteristics	Reference
*E. coli*		
DH5α	F^−^Φ80*dlac*Δ(*lacZ*)M15 Δ(*lacZYA-argF*) U169 *endA1 recA1 hsdR17* (r_K_^−^ m_K_^+^) *deoR thi-1 phoA supE44 λ*^−^ *gyrA96 relA1*; strain used for cloning procedures	[61]
BL21(DE3)	F-*ompT hsdS*_B_ (r_B_-, m_B_-) *gal dcm* (DE3); host for protein production	[69]
*C. glutamicum*		
ATCC13032 (WT)	Biotin-auxotrophic wild type	[70]
WT Mrp1C_G29D_	ATCC13032 with mutation Mrp1C_G29D_ (Cg0325)	This work
C1*	Derivative of ATCC13032 with a genome reduced by 13.4%	[56]
C1*ΔLEU ARG^+^::P_*tac*_-*eyfp* (ΔLEU ARG^+^)	C1*ΔLEU ARG^+^ with *eyfp* under control of P_*tac*_ integrated into the IGR between cg1121 and cg1122	[9]
WTΔARG LEU^++^::P_*tac*_-*crimson* (ΔARG LEU^++^)	WTΔARG LEU^++^ with *crimson* under control of P_*tac*_ integrated in the IGR between cg1121 and cg1122	[9]
C1*ΔLEU ARG^++^	C1* ΔLEU ARG^+^ with point mutations ArgB_A26V M31V_ (Cg1582)	[9]
C1*ΔLEU ARG^++^::P_*tac*_-*eyfp* (ΔLEU ARG^++^)	C1*ΔLEU ARG^++^ with *eyfp* under control of P_*tac*_ integrated into the IGR between cg1121 and cg1122	This work
ΔLEU ARG^+^ evo1	Derivative of ΔLEU ARG^+^ isolated after evolution. For details about identified mutations, see Table 1	This work
ΔLEU ARG^+^ evo2	Derivative of ΔLEU ARG^+^ isolated after evolution. For details about identified mutations, see Table 1	This work
ΔARG LEU^++^ evo1	Derivative of ΔARG LEU^++^ isolated after evolution. For details about identified mutations, see Table 1	This work
ΔARG LEU^++^ evo2	Derivative of ΔARG LEU^++^ isolated after evolution. For details about identified mutations, see Table 1	This work
ΔARG LEU^++^ evo3	Derivative of ΔARG LEU^++^ isolated after evolution. For details about identified mutations, see Table 1	This work
C1*ΔLEU ARG^+^::P_*tac*_-*eyfp* MetC/P_brnQ_* (ΔLEU ARG^+^ MetC/P_brnQ_*)	C1*ΔLEU ARG^+^::P_*tac*_- *eyfp* with mutation MetC_S322F_ (Cg2536), which is also within the promoter region of *brnQ* (cg2537)	This work
C1*ΔLEU ARG^+^::P_*tac*_-*eyfp* Mrp1C_G29D_ (ΔLEU ARG^+^ Mrp1C_G29D_)	C1*ΔLEU ARG^+^::P_*tac*_- *eyfp* with mutation Mrp1C_G29D_ (Cg0325)	This work
C1*ΔLEU ARG^+^ Mrp1A_H335P_ (ΔLEU ARG^+^ Mrp1A_H335P_)	C1*ΔLEU ARG^+^::P_*tac*_- *eyfp* with mutation Mrp1A_H335P_ (Cg0326)	This work
C1*ΔLEU ARG^+^::P_*tac*_-*eyfp* MetC/P_brnQ_* Mrp1C_G29D_ (ΔLEU ARG^+^ MetC/P_brnQ_* Mrp1C_G29D_)	C1*ΔLEU ARG^+^::P_*tac*_- *eyfp* MetC/P_brnQ_* with mutation Mrp1C_G29D_ (Cg0325)	This work
C1*ΔLEU ARG^+^::P_*tac*_-*eyfp* Δ*mrp1* (ΔLEU ARG^+^ Δ*mrp1*)	C1*ΔLEU ARG^+^::P_*tac*_- *eyfp* with deletion of *mrp1* (cg0321-cg0326) and cg0317-cg0319. The latter encode genes for arsenate/arsenite resistance and were deleted accidentally	This work
WTΔARG LEU^++^::P_*tac*_-*crimson* P_argT_*^1^ (ΔARG LEU^++^ P_argT_*^1^)	WTΔARG LEU^++^::P_*tac*_-*crimson* with mutation A ➜ G 35 bp upstream of the *argT* (cg1504) TSS/TLS	This work
WTΔARG LEU^++^::P_*tac*_-crimson argT* (ΔARG LEU^++^ argT*)	WTΔARG LEU^++^::P_*tac*_-*crimson* with mutation of the 3^rd^ codon of *argT* (cg1504) GAG ➜ GAA, synonymous mutation	This work
WTΔARG LEU^++^::P_*tac*_-*crimson* P_argT_*^1^ Cg1874_G93D_ (ΔARG LEU^++^ P_argT_*^1^ Cg1874_G93D_)	WTΔARG LEU^++^::P_*tac*_-*crimson* P_argT_*^1^ with mutation Cg1874_G93D_	This work
WTΔARG LEU^++^::P_*tac*_-*crimson* P_argT_*^1^ Cg1874_G93D_ Cg2850_G30R_ (ΔARG LEU^++^ P_argT_*^1^ Cg1874_G93D_ Cg2850_G30R_)	WTΔARG LEU^++^::P_*tac*_-*crimson* P_argT_*^1^ Cg1874_G93D_ with mutation Cg2850_G30R_	This work
WTΔARG LEU^++^::P_*tac*_-*crimson* Δ*argTUV* (ΔARG LEU^++^ Δ*argTUV*)	WTΔARG LEU^++^::P_tac_-crimson with in frame deletion of *argTUV* (cg1504-1502)	This work
WTΔARG LEU^++^::P_*tac*_-*crimson* Δcg1505-1506 (∆ARG LEU^++^ ∆cg1505-cg1506)	WTΔARG LEU^++^::P_tac_-crimson with deletion of cg1505 and cg1506 including their promoters	This work
WT∆LEU ARG^−+^::P_*tac*_-*eYFP* (∆LEU ARG^−+^)	WT with an in-frame deletion of Δ*leuA* (cg0303), Δ*leuC* (cg1487), Δ*leuD* (cg1488), Δ*leuB* (cg1453), with point mutations ArgB_A26V M31V_ (Cg1582) and *eyfp* under control of P_*tac*_ integrated into the IGR between cg1121 and cg1122	This work

### CoNoS evolution

Adaptive laboratory evolution (ALE) was performed using the Mini Pilot Plant described in previous work [15, 16]. In brief, three wells of a 48-well Flowerplate were used to cultivate the CoNoS of interest in CGXII medium in the BioLector. Cell growth was monitored with online backscatter measurement until a backscatter threshold triggered automated cell suspension aliquot transfer to an empty well that was immediately filled with chill-stored CGXII medium for the next batch. In this way, 16 repetitive batches were performed in biological triplicates. Process modelling was performed to estimate specific growth rates for each single batch of the ALE experiment. The model was setup in OpenModelica [59] and validated using the in-house python-based package Estim8 (unpublished). From material of the last batches, single strains were isolated and retested with a non-evolved partner in a CGXII culture in the BioLector.

### Microscale cultivation

The fitness of single strains and co-cultures was investigated using a micro bioreactor with online backscatter measurement. All strains were cultivated as described [9]. In brief, each strain was spread from a cryo stock onto BHI plates. Single colonies were used to inoculate a preculture in amino acid-supplemented CGXII medium with 2% (w/v) glucose and cultivated for two days at 30 °C and 250 rpm. Afterwards, the precultures were centrifuged, the pellet was suspended in sterile 0.9% (w/v) NaCl and used to inoculate the main cultures. The main cultures were cultivated in CGXII or CGXII supplemented with amino acids in 48-well Flowerplates (m2p-labs GmbH, Germany) in a BioLector system (m2p-labs GmbH, Germany) at 1400 rpm, 85% humidity and 30 °C. Co-cultures were inoculated in a 1:1 ratio of the two strains. Growth rate evaluation was performed using the Python package Bletl [60] as described [9]. For characterizing the substrate uptake and amino acid production, automated harvesting and processing of cultures was performed using the Mini Pilot Plant and resulting cell-free supernatants were analyzed via HPLC.

### Amino acid quantification by HPLC

Amino acids were separated and quantified on an uHPLC system (Agilent 1290 Infinity, Agilent Technologies, Santa Clara, CA). 50 mM α-Aminobutyric acid (AABA) was added as internal standard to the properly diluted cell-free supernatants. A precolumn (Phenomenex, SecurityGuard™ ULTRA C18, sub-2 µm, 2.1 mm internal diameters) and a reverse phase column (Kinetex 2.6 µm EVO C18 100 Å, 100 × 2.1 mm) were used as stationary phase. In the mobile phase, buffer A (10 mM Na_2_HPO_4_ (anhydr.), 10 mM Na_2_B_4_O_7_ × 10 H_2_O, pH 8.2 with HCl) and buffer B (methanol) with a flow rate of 0.42 mL min^−1^ were used with a column temperature of 40 °C and an injection volume of 1 μL. Precolumn derivatization with ophthaldialdehyde (OPA) reagent (Sigma-Aldrich, ready-to-use mix) was performed in an automated procedure. 2 μL OPA, 1 μL sample and 2 μL water were mixed in the injection loop six times and incubated for 1 min. Amino acids were separated with the following elution conditions (min/B%): 0.0 min/2%; 0.5 min/2% to 20.0 min/57%; 20.1 min/100%; 23.6 min/2%; 25.0 min END. Amino acids were detected using a fluorescence detector (Agilent 1290 FLD) with an excitation wavelength of 340 nm and an emission wavelength of 450 nm. Target amino acids were quantified relatively to amino acid standards of known concentrations measured before and after each run and to the internal standard.

### Recombinant DNA work

In this work, *Escherichia coli* DH5α [61] was used as host for molecular cloning. All plasmids used in this study are listed in Additional file 1: Table S4 and all oligonucleotides in Additional file 1: Table S5. Deletions and mutations in *C. glutamicum* were introduced via the pK19*mobsacB*-system as described previously [57, 62].

### DNA isolation and sequencing

For DNA isolation, single strains were grown in a CGXII monoculture supplemented with 3 mM of the respective amino acid in the BioLector. From one well per mutant, gDNA was isolated with the DNeasy Blood & Tissue Kit (Qiagen, Hilden, Germany). Resulting gDNA concentration was determined via Qubit 2.0 fluorometer (Thermo Fisher Scientific, Waltham, USA). From the prepared gDNA, 1 µg was used for library preparation employing the NEBNext® Ultra™ II DNA Library Prep Kit (NEB, Frankfurt am Main, Germany). Via qPCR with the KAPA library quantification kit (Peqlab, Erlangen, Germany), the library was evaluated and then normalized via pooling. After in-house sequencing (paired-end sequencing via a MiSeq (Illumina®), read length of 2 × 150 bases), the demultiplexed fastq output files were processed with the CLC Genomic Workbench software (Qiagen, Hilden, Germany). For reads mapping and variants calling, the *C.* *glutamicum* ATCC 13032 reference genome BX927147 or the genome sequence of *C. glutamicum* C1 (CP017995) were used. Mutations and deletions were assessed manually regarding their specific occurrence between the different samples and their relevance. The data for this study have been deposited in the European Nucleotide Archive (ENA) at EMBL-EBI under accession number PRJEB60176 (https://www.ebi.ac.uk/ena/browser/view/PRJEB60176).

### Analysis of gene expression levels

Precultures of 5 mL BHI media in a test tube were inoculated with a single colony from a BHI plate and incubated for seven hours at 30 °C and 170 rpm. The cells were centrifuged for 5 min at 1700*g* and washed once in PBS (phosphate buffered saline, 137 mM NaCl, 2.7 mM KCl, 10 mM Na_2_HPO_4_, 1.8 mM KH_2_PO_4_, pH 7.4 with HCl). Subsequently, the cells were suspended in CGXII medium with 2% (w/v) d-glucose and 3 mM of the appropriate amino acid supplementation and incubated in test tubes at 30 °C and 170 rpm overnight. For the main cultures, 50 ml CGXII media with 2% (w/v) d-glucose and 3 mM of the appropriate amino acid supplementation were inoculated to an OD_600_ of 0.5 in a 500 mL baffled flask and incubated at 30 °C and 130 rpm in a Minitron shaker (Infors HT, Einsbach, Germany) until an OD_600_ of 5 was reached. 25 ml cell suspension was mixed with 25 g ice and centrifuged for 10 min at 3720*g* and 4 °C. The supernatant was removed and the cell pellets were frozen in liquid nitrogen and stored at − 80 °C or used immediately.

For RNA isolation, the RNeasy MiniKit (QIAGEN, Hilden, Germany) was used. The pellets were suspended in 700 μL RLT buffer and transferred into two Precellys® tubes with 250 mg glass beads. The tubes were set into a Prececllys®24 tissue homogenizer (Bertin GmbH, Frankfurt, Germany) and the cells were disrupted with two cycles at 6000 rpm, with 20 s per cycle and stored on ice in between runs. The tubes were centrifuged at 21,300* g* at room temperature for 2 min. The supernatant was transferred to a fresh tube and mixed with 250 μL ethanol (-20 °C). The mixture was added to an RNeasy Mini-Kit Spin column and centrifuged for 15 s at 12,633*g* and room temperature. Afterwards, the protocol provided by the manufacturer was followed including DNAase on column digest. The purified RNA was diluted 1:10 with ddH2O and the RNA concentration was measured at 260 nm with a Colibri Microvolume Spectrometer (Titertek-Berthold, Germany).

All reverse transcriptase quantitative PCRs (RT-qPCRs) were prepared with the New England Biolabs® Inc. Luna® Universal Probe One-Step RT-qPCR Kit. SYBR®Green was used as probe. For each reaction, the reaction mix with a volume of 20 μL was prepared according to the instructions from the manufacturer. The PCR plate was covered with a foil to prevent evaporation. The plate was centrifuged for 1 min at 665*g* at room temperature. The PCR plate was placed in the PCR cycler qTOWER 2.2 (Analytic Jena, Germany) and incubated with the following program: 55 °C for 10 min, 95 °C for 1 min, 40 times (95 °C for 10 s, 60 °C for 30 s) followed by a melt curve 60–95 °C with 6 s for ΔT = 1 °C. For each qPCR, a standard dilution was prepared for each RNA used in the qPCR. Duplicate 20 µl reactions were prepared containing 500 ng, 50 ng, 5 ng, 0.5 ng or 0.05 ng RNA of the native ΔLEU ARG^+^ or ΔARG LEU^++^ strains in nuclease-free water. For all other samples, 300 ng of template RNA were used. As reference gene, meso-diaminopimelate dehydrogenase (*ddh*, cg2900) was used with the oligonucleotides listed in Additional file 1: Table S5, resulting in a 150 bp product. Samples with *ddh* primers were prepared for each RNA used. Primers were designed with Primer3Plus [63]. For all samples, two biological replicates with six technical replicates each were measured. For data analysis, the qPCR software qPCR 3.1 (Analytik Jena, Germany) and the Livak method [64] were used to determine the 2^ΔΔCt^ value.

### Prediction of the impact of mutations on protein structures

The MSA-based ab initio prediction of monomeric structures for *C. glutamicum* MetC, Cg1874, and Cg2850 WT proteins as well as of their mutated variants (MetC_S322F_, Cg1874_G93D_, Cg2850_G30R_) was performed using AlphaFold2 via the ColabFold pipeline [65, 66] applying mostly default parameters (use_amber: no, template mode: none, msa_mode: MMSeq2 (UniRef + Environmental), num_recycle: 3). The resulting predicted structures with the highest model confidence (based on pLDDT and predicted aligned error (PAE) confidence measures) for each protein were analyzed for structural changes using ChimeraX [67].

### Protein production and purification

His-tagged ArgT was overproduced using *E. coli* BL21(DE3) pET-TEV-*argT*. The strain was cultivated at 37 °C in terrific broth (TB) [68]. After induction of target gene expression with 500 µM IPTG, the cells were cultivated for 18 h at 18 °C. Cells were harvested by centrifugation for 20 min at 5500*g* and suspended in lysis buffer (20 mM Tris–HCl pH 7.9, 500 mM NaCl, 5% (v/v) glycerol, 20 mM imidazole,) containing cOmplete EDTA-free protease inhibitor (Roche, Basel, Switzerland), and disrupted by Multi Shot high-pressure homogenizer (Constant systems Ltd., Daventry, United Kingdom) treatment at 20,000 psi. Soluble protein fractions were obtained by centrifugation (5000*g*, 4 °C, 20 min) and subsequent ultracentrifugation of the supernatant (100,000*g*, 4 °C, 1 h). Supernatants of the ultracentrifugation were loaded onto a HisTrap HP column (GE Healthcare, Chicago, IL, USA) and, after washing, the His-tagged protein was eluted using lysis buffer with increasing imidazole concentrations up to 300 mM. The protein was further purified by size exclusion chromatography on a Superdex 200 10/300 GL column (GE Healthcare, Chicago, IL, USA) equilibrated in HEPES buffer (40 mM HEPES-NaOH, 100 mM NaCl, pH 7.4). Protein concentrations were determined using a Colibri microvolume spectrometer (Berthold Detection Systems GmbH, Pforzheim, Germany) and the molar extinction coefficient was predicted by the ProtParam tool (http://web.expasy.org/protparam/).

### Isothermal titration calorimetry

Purified His_10_-ArgT was dialyzed overnight in HEPES buffer (40 mM HEPES–NaOH, pH 7.4, 100 mM NaCl). 20 mM stock solutions of the potential ligands were prepared in dialysis buffer, and the pH was adjusted to pH 7.4 using NaOH or HCl. ITC measurements were performed with a MicroCal PEAQ-ITC instrument (Malvern Panalytical, Malvern, United Kingdom) operated at 25 °C. Protein concentrations of 30 μM and ligand concentrations of 50 µM to 2 mM were used. Prior to filling the measuring cell with 300 μL protein solution, the cell was rinsed with dialysis buffer, and the syringe was filled with 75 μL ligand solution. An ITC run was started with an initial injection of 0.4 μL followed by 18 injections of 2 μL each. In addition, control experiments with ligand solution titrated into the dialysis buffer were performed. The data were analyzed using MicroCal ITC analysis software (Malvern Panalytical, Malvern, United Kingdom).

## Supplementary Information

**Additional file 1.** Tables S1–S5, Figures S1–S15.

## Data Availability

The genome sequencing data generated during this study has been deposited in the European Nucleotide Archive (ENA) at EMBL-EBI under accession number PRJEB60176 (https://www.ebi.ac.uk/ena/browser/view/PRJEB60176). All other data generated or analysed during this study are included in this published article and its supplementary information files. Strains and plasmids generated during this study are available from the corresponding author upon request.
