# Supplementary material for: Discovery of novel amino acid production traits by evolution of synthetic co-cultures
Source: Microb Cell Fact. 2023 Apr 15;22:71. doi: 10.1186/s12934-023-02078-2 (PMC10105947; doi:10.1186/s12934-023-02078-2)
Supplement: Supplementary file 1 — Additional file 1. Tables S1–S5, Figures S1–S15. [file 12934_2023_2078_MOESM1_ESM.pdf]

Supplement to:

Discovery of novel amino acid production traits by evolution of synthetic co-cultures

Rico Zuchowski[1#], Simone Schito[1#], Friederike Neuheuser[1], Philipp Menke[1], Daniel Berger[1], Niels Hollmann[1], Srushti Gujar[1,2,3], Lea Sundermeyer[1], Christina Mack[1], Astrid Wirtz[1], Oliver H. Weiergräber[2], Tino Polen[1], Michael Bott[1], Stephan Noack[1], Meike Baumgart[1,*]

[1]Institute of Bio- and Geosciences, IBG-1: Biotechnology, Forschungszentrum Jülich, Jülich, Germany

[2]Institute of Biological Information Processing, IBI-7: Structural Biochemistry, Forschungszentrum Jülich, Jülich, Germany

[3]Institut für Physikalische Biologie, Heinrich-Heine-Universität Düsseldorf, Düsseldorf, Germany

[#]These authors contributed equally to this work

*Address correspondence to m.baumgart@fz-juelich.de

Supplemental tables

Table S1: Amino acid importer in *C. glutamicum*

Locus tag(s)	NCgl synonyms	Transporter name	Amino acid substrate	Reference
cg0606-cg0610	NCgl0497-NCgl0501	MetQNI	L-methionine	(1)
cg1105	NCgl0929	LysI	L-lysine	(2)
cg1167-cg1169	NCgl0985-NCgl0986	MetPS	L-methionine<br>L-alanine	(1)
cg1257	NCgl1062	AroP	L-phenylalanine<br>L-tryptophan<br>L-tyrosine<br>L-histidine	(3, 4)
cg1305	NCgl1108	PheP	L-phenylalanine<br>L-histidine	(5, 6)
cg1314	NCgl1116	PutP	L-proline	(7)
cg1502-cg1504	NCgl1276-NCgl1278	ArgTUV	L-arginine	This study
cg2136-cg2139	NCgl1875-NCgl1878	GluABCD	L-glutamate	(8)
cg2537	NCgl2228	BrnQ	L-leucine<br>L-valine<br>L-isoleucine	(9, 10)
cg2539	NCgl2230	EctP	L-proline	(11)
cg2810	NCgl2643	CynT	L-cysteine	(12)
cg3396	NCgl2962	ProP	L-proline	(11)

Table S2: Mean pLDDT and C^{α} r.m.s.d. values for WT and mutant structures predicted in this study.

Structures predicted by AlphaFold2 (rank 1)	Mean pLDDT (0–100)	C^{α} r.m.s.d. of WT and mutant
MetC	97.96	0.26 Å
$MetC_{S322F}$	97.84	
Cg1874	94.52	0.13 Å
$Cg1874_{G93D}$	94.69	
Cg2850	89.13	0.21 Å<br>(excluding disordered residues 1–27)
$Cg2850_{G30R}$	89.46	

Table S3: Ligands tested for binding to ArgT via ITC measurements.

Tested amino acids	Binding observed?	Tested concentrations
L-arginine	yes	50 µM - 200 µM
L-citrulline	yes	200 µM, 100 µM
L-cysteine	no	200 µM, 2 mM
L-glutamate	no	200 µM, 2 mM
L-glutamine	no	200 µM, 2 mM
L-histidine	no	200 µM, 2 mM
L-lysine	no	200 µM, 2 mM

Table S4: Plasmids used in this study.

Plasmid	Relevant characteristics	Source or reference
pK19*mobsacB*	Kan^r; plasmid for allelic exchange in *C. glutamicum*; (pK18 $oriV_{E.c.}$, *sacB*, *lac*Zα)	(13)
pK19*mobsacB*-$ArgB_{A29V\ M31V}$	Kan^r; pK19*mobsacB* derivative for mutation of *argB* (cg1582) to yield $ArgB_{A29V\ M31V}$ in *C. glutamicum*	(14)
pK19*mobsacB*-$MetC_{S322F}/P_{brnQ}$*	Kan^r; pK19*mobsacB* derivative for mutation of MetC (Cg2536) S322F in *C. glutamicum.* This mutation also affects the promoter region of *brnQ* (cg2537).	This study
pK19*mobsacB*-$Mrp1C_{G29D}$	Kan^r; pK19*mobsacB* derivative for mutation of Mrp1C (Cg0325) G29D in *C. glutamicum*	This study
pK19*mobsacB*-$Mrp1A_{H335P}$	Kan^r; pK19*mobsacB* derivative for mutation of Mrp1A (Cg0326) H335P in *C. glutamicum*	This study
pK19*mobsacB*-P_{argT}*[1]	Kan^r; pK19*mobsacB* derivative for the mutation A→G 35 bp upstream of the *argT* (cg1504) TSS/TLS	This study
pK19*mobsacB*-*argT**	Kan^r; pK19*mobsacB* derivative for mutation of the 3rd codon of *argT* (cg1504) GAG→GAA, synonymous mutation	This study
pK19*mobsacB*-Δ*argTUV*	Kan^r; pK19*mobsacB* derivative for in frame deletion of *argTUV* (cg1504-cg1502) in *C. glutamicum*	This study
pK19*mobsacB*-Δcg1505-cg1506	Kan^r; pK19*mobsacB* derivative for in frame deletion of cg1505-cg1506 in *C. glutamicum*	This study
pK19*mobsacB*-Δ*mrp1*	Kan^r; pK19*mobsacB* derivative for deletion of *mrp1* (cg0321-cg0326) and cg0317-cg0319 in *C. glutamicum*. The latter encode genes for arsenate/arsenite resistance and were deleted accidentally.	This study
pPREx2	Kan^r; *E. coli/C. glutamicum* shuttle vector for expression of target genes. Cured pEKEx2-derivative with corrected *lacI*^{q} and without replicative sequences. P_{tacI}; *lacI*^{q}; $ori_{C.g.}$ of pBL1; $ori_{E.c.}$ ColE1 of pUC18.	(15)
pPREx2-*argTUV*	Kan^r; pPREx2 derivative with the *argTUV* genes (cg1504-cg1502) under control of P_{tac} promoter	This study
pET-TEV	Kan^R; pET28b derivative for overexpression of genes in *E. coli*, adding an N-terminal decahistdine tag and a TEV protease cleavage site to the target protein (pBR322 $oriV_{E.c.}$, PT7, lacI)	(16)
pET-TEV-*argT*	Kan^R; pET-TEV derivative coding for the ArgT protein (Cg1504) with an N-terminal decahistdine tag and a TEV protease cleavage site	This study

Table S5: Oligonucleotides used in this study.

Oligonucleotide	Sequence (5' → 3')[a]
Sequencing primers	
B223_M13-fw	CGCCAGGGTTTTCCCAGTCAC
B224_M13-rv	AGCGGATAACAATTTCACACAGGA
Verification of deletion of cg3035	
B463_cg3035_Dfw	GCGGAAAGCATGCTTAGAATGTTGCC
B464_cg3035_Drv	CACGTTCTGGTTCGGTGACG
Work with pK19*mobsacB*-Δ*arg*	
B469_ARG-Dfw	AGCTCCCGCTAAGGTAGCTACC
B470_ARG-Drv	TTGGGTTTCATTCAACAACGCGCC
Verification of deletion of *leuA*	
Z5_leuA-Dfw	GGGTGGATTCCACTTGATTG
Z6_leuA-Drv	GGCTACCCTCCTCACCGTAG
Verification of deletion of *leuB*	
Z11_leuB-Dfw	GGTGCTTAAGGCGGATAAGG
Z12_leuB-Drv	TAAACACCCTCGGTGCTCTG
Verification of deletion of *leuCD*	
Z17_leuCD-Dfw	CCCACGGACTCCGCTAAGC
Z18_leuCD-Drv	TCAAACCCACCGCAATTTACTCG
Verification of deletion of *argR*	
Z55_ArgR-Dfw	ACTGTGCCGCTGGTGAACTC
Z56_ArgR-Drv	AGTCACGAGCAGGTGCAATG
Work with pK19*mobsacB*-$ArgB_{A29V\ M31V}$	
Z78_ArgB_mut-fw_neu	TGTGACCAAGCGCGTTGCTG
Z64_ArgB-mut_rv	GCCGTCAATGACATGAGCAG
Verification of P_{tuf} *leuA*_B018_BS	
Z92_LeuA_Ptuf_fw	CTGGACTTCGTGGTGGCTAC
Z93_LeuA_Ptuf_seq_rv	TGCCACAGGGTAGCTGGTAG
Z67_PtufLeuA_seq_fw	CTCAGTGGTGTGCTGTTGAC
Z68_PtufLeuA_seq_rv	TCCTTGCCGTTGTGGATGAG
Construction and work with pK19*mobsacB*-$MetC_{S322F}$	
n071_cg2536_D1	**AAAACGACGGCCAGTGAATT**GATTCCACGACAACGCC
n072_cg2536_D2	GAGGCGCCTTTTAAAATTCTTCG
n073_cg2536_D3	**GAATTTTAAAAGGCGCCTCT**TCGATCTTG
n074_cg2536_D4	**CAGGTCGACTCTAGAGGA**GCTGACGGTGCGGGATC
n021_cg2536_Dfw	TGCCTGCATACCCTCCTTTC

Oligonucleotide	Sequence (5' → 3')[a]
n022_cg2536_Drv	AACTTGGCTACCGACAACAC
Construction of and work with pK19*mobsacB*-Mrp1C$_{G29D}$	
n023_cg0325_D1	**AAAACGACGGCCAGTGAATT**GCTCCTGTACCTGTCCAA
n024_cg0325_D2	TCAGTGACATGTCGAAGACGATGC
n025_cg0325_D3	**CGTCTTCGACATGTCACTGA**TCGGCCACGCAGCGAACTTG
n026_cg0325_D4	**CAGGTCGACTCTAGAGGA**GGGGATTGCCACGCCACCGACA
n051_cg0325_Dfw	CACCGACGATGGTGGCAAAC
n052_cg0325_Drv	GCGCTATGCTTCTTGAGCTTC
Construction of and work with pK19*mobsacB*-Mrp1A$_{H335P}$	
n027_cg0326_D1	**AAAACGACGGCCAGTGAATT**CGCTGCTTACTGCGGTGGCG
n028_cg0326_D2	CTTAAACAGCGCGGGGCTGAGCGTG
n029_cg0326_D3	**CAGCCCCGCGCTGTTTAAG**TCCTCGTTGTTCATGCTC
n030_cg0326_D4	**CAGGTCGACTCTAGAGGA**GTGGGGTGTTGATGCCGTGCC
n053_cg0326_Dfw	ACCAGCATCGATAGGATGTC
n054_cg0326_Drv	TGTTGATTGCGCGTTCGG
Construction of and work with pK19*mobsacB*-P$_{argT}$*[1]	
n039_cg1504_1399043_D1	**AAAACGACGGCCAGTGAATT**CTTGTGCGGGTGATACTCC
n040_cg1504_1399043_D2	GCAATAACCTCAAGTGAGGC
n041_cg1504_1399043_D3	**CTCACTTGAGGTTATTGC**ACCATGGCAGGTAG
n042_cg1504_1399043_D4	**CAGGTCGACTCTAGAGGA**GTTCTCGCGGCGTTCCTCG
n057_cg1504_Dfw	ACGGACATCGTCGGTCTCTG
n058_cg1504_Drv	CTGCATTTGGATCTGCCTGTTG
Construction of and work with pK19*mobsacB-argT**	
n035_cg1504_1399000_D1	**AAAACGACGGCCAGTGAATT**CCCAACGATCACTTCCACTC
n036_cg1504_1399000_D2	CGGGTGAAACTTTCAATCATGG
n037_cg1504_1399000_D3	**GATTGAAAGTTTCACCCG**ATTGCGAATTTCGCAGACAC
n038_cg1504_1399000_D4	**CAGGTCGACTCTAGAGGA**GGTTTCACGATCAGTTGCCTGC
n057_cg1504_Dfw	ACGGACATCGTCGGTCTCTG
n058_cg1504_Drv	CTGCATTTGGATCTGCCTGTTG
Construction of and work with pK19*mobsacB*-Δ*argTUV*	
n043_cg1502-1504_D1	**AAAACGACGGCCAGTGAATT**CTGCCTCTTCCACTTGTGC
n044_cg1502-1504_D2	**AGCCGATAATGACTCGCA**GCAATAACCTTAAGTGAGGC
n045_cg1502-1504_D3	**CTTAAGGTTATTGC**GCGAGTCATTATCGGCT
n046_cg1502-1504_D4	**CAGGTCGACTCTAGAGGA**GACACGTGAAATAAGGAGG
n059_cg1502-1504_Dfw	AAGAAGTTGCCCGCTTCGGTG
n060_cg1502-1504_Drv	CTGCATTTGGATCTGCCTGTTG

**Oligonucleotide**	**Sequence (5’ → 3’)**[a]
Construction of and work with pK19*mobsacB*-Δcg1505-1506	
n047_cg1505-1506_D1	**AAAACGACGGCCAGTGAATT**GCACCACTACCAGCG
n048_cg1505-1506_D2	**GTTTTATCCGGCATGGG**CCAATTATCTTTCTTTGGCG
n049_cg1505-1506_D3	**CAAAGAAAGATAATTGG**CCCATGCCGGATAAAAC
n050_cg1505-1506_D4	**CAGGTCGACTCTAGAGGA**GCTTGAACCGATGGAAGG
n061_cg1505-1506_Dfw	ACGGACATCGTCGGTCTCTG
n062_cg1505-1506_Drv	TACTCGGCCATGCTGACTCAC
Construction of and work with pK19*mobsacB*-Δ*mrp1*	
n031_cg0322-0326_D1	**AAAACGACGGCCAGTGAATT**CTGTACAACGACAAGACCGCC
n032_cg0322-0326_D2	**CTGCAATAGAATGTGTTC**CTACTTGATGAGTTAGCAAGAGCAC
n033_cg0322-0326_D3	**CTTGCTAACTCATCAAGTAG**GAACACATTCTATTGCAGGG
n034_cg0322-0326_D4	**CAGGTCGACTCTAGAGGA**GCACCTTGTTGAGCGTTG
n055_cg0322-0326_Dfw	ACGTTTCGCAGCAATGCGATAG
n056_cg0322-0326_Drv	TGTTTGCGCAGGTCTTCGG
Primer for gene expression analysis via RT-qPCR	
n083_ddh_qPCR_fw	CCGGAAAGCAAACCCACAAG
n084_ddh_qPCR_rev	CTCGGAGTCGAAGGTTGCTT
n085_cg1502_qPCR_fw	GCAAGTCAATGCCCTTGAGC
n086_cg1502_qPCR_rv	CTGATGCTGAAGGCGCAATC
n087_cg1504_qPCR_fw	ACGAGGTCCATTTCCACACC
n088_cg1504_qPCR_rv	CGTTCCAGATCCTGTACCGG
n089_cg2537_qPCR_fw	AAGGACATGGCTTCTCGTGG
n090_cg2537_qPCR_rv	AACAAGCCCGAATAAAGCGC
Z315_cg2538_qPCR_fw	TCGCCAAATTGTCGACATGC
Z316_cg2538_qPCR_rv	ATATTGCGCGTGGTTGCTTC
Analysis of fluorescent protein integration	
Z109_Intcg1121 fwd	TTGGCGTGTGGTTGGTTAG
Z110_Intcg1122 rev	CGCATCAAGCAGATCTCTG
Construction of and work with pK19*mobsacB*-Cg1874$_{G93D}$	
n003_cg1874_Dfw	TAAATTGCGGGTGCTGTTGG
n004_cg1874_Drv	CTGCAAGCAGCACTGGATCG
n013_cg1874_mutate-D1	**AAAACGACGGCCAGTGAATT**ACTAAAGCTTGGGCAGCGAC
n014_cg1874_mutate-D2	CCGGCAACGAGGTCCTTCTTGTC
n015_cg1874_mutate-D3	**AAGAAGGACCTCGTTGCCGG**CGGATGGCGCTTTTCAGATC
n016_cg1874_mutate-D4	**CAGGTCGACTCTAGAGGA**CTGAGGCAATTCCCCTGGAA

Oligonucleotide	Sequence (5’ → 3’)[a]
Construction of and work with pK19*mobsacB*-Cg2850$_{G30R}$	
n007_cg2850_Dfw	AACCGCGAACCAACGAGTCC
n008_cg2850_Drv	CCGCTAGTGCGGTATCGAAC
n017_cg2850_mutate-D1	**AAAACGACGGCCAGTGAATT**CTTCCGAATCAACTCCACCA
n018_cg2850_mutate-D2	TGCGTCGGATCGGCTGATGCTTG
n019_cg2850_mutate-D3	**GCATCAGCCGATCCGACGCA**GTAAACCTCGCTGCCGAACA
n020_cg2850_mutate-D4	**CAGGTCGACTCTAGAGGA**AGTTCGTTGGTTGGAAGCAG
Construction of pET-TEV-*argT*	
DB029_pET-TEV_cg1504_AS78_D2	**ACCTGTATTTTCAGGGCCAT**CCCGAAGCTCTGGCTCAGCG
DB030_pET-TEV_cg1504_D3_rev	**AAGCTTGTCGACGGAGCTCG**CTAGTTGAGTGGCTGTTCGTTG

[a] Overhangs for Gibson assembly (17) are marked with bold letters.

Supplemental figures

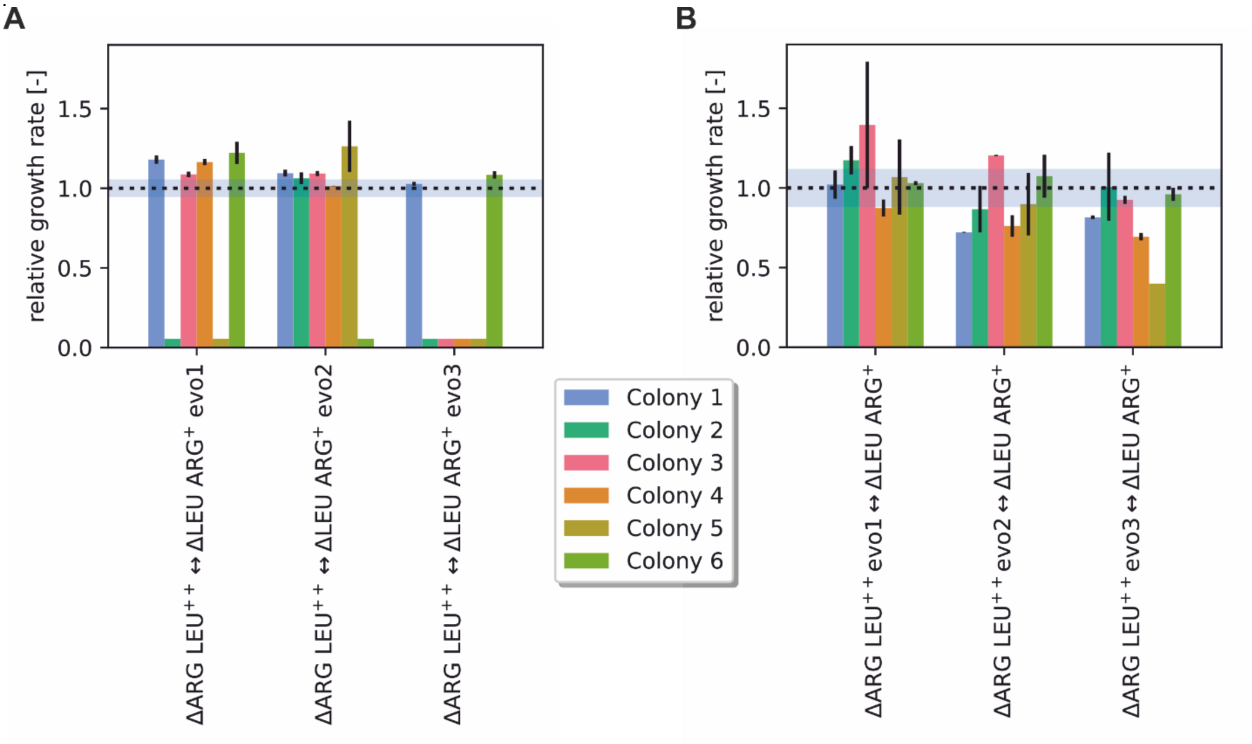

Fig. S1: Growth rates of different CoNoS established using colonies isolated from an ALE experiment. Single auxotrophic CoNoS member were isolated on CGXII plates supplemented with the required amino acid and six colonies from each ALE replicate were tested in combination with the non-evolved partner in a CoNoS setup. The dotted line and the corresponding blue shady background indicate the relative growth rate and the standard deviation of the non-evolved CoNoS as reference cultivation. Control CoNoS were cultivated in biological triplicates, test CoNoS were cultivated in technical duplicates. (A) Test of colonies derived from evolved *C. glutamicum* ΔLEU ARG^{+}. (B) Test of colonies derived from evolved *C. glutamicum* ΔARG LEU^{++}.

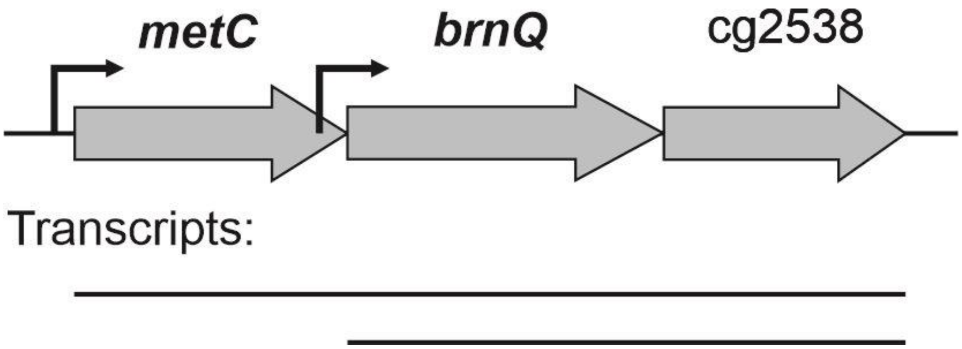

Fig. S2: Genomic organization of the region near *metC* and *brnQ*. Black arrows represent transcriptional start sites. Black lines in the lower part of the figure represent potential operons and sub operons. Transcript and promoter data based on published RNAseq data (18).

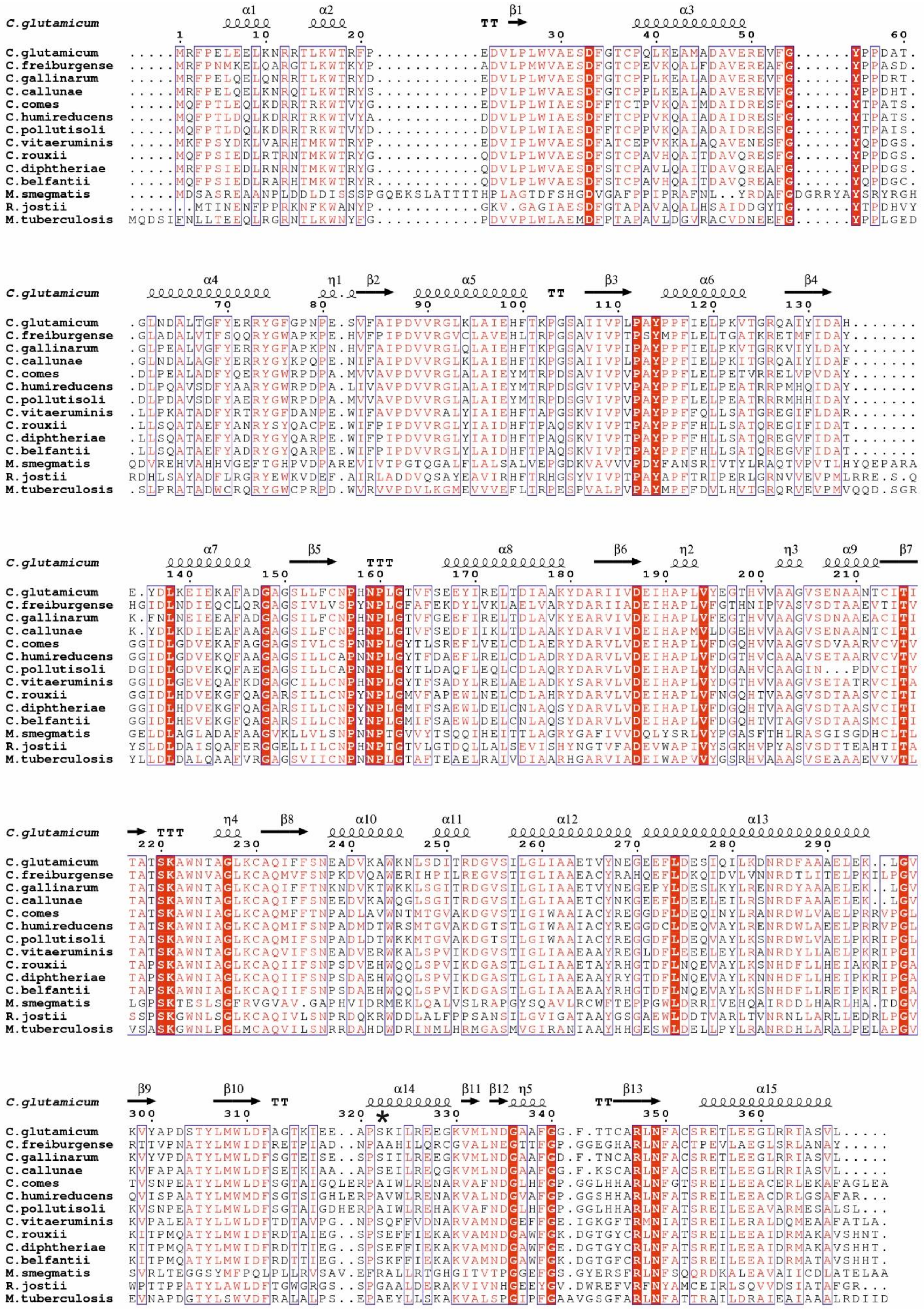
C.glutamicum
C.freiburgense
C.gallinarum
C.callunae
C.comes
C.humireducens
C.pollutisoli
C.vitaeruminis
C.rouxii
C.diphtheriae
C.belfantii
M.smegmatis
R.jostii
M.tuberculosis
α1 α2 β1 α3 α4 η1 β2 α5 β3 α6 β4 α7 β5 α8 β6 η2 η3 α9 β7 η4 β8 α10 α11 α12 α13 β9 β10 α14 β11 β12 η5 β13 α15
TT
TTT

Fig. S3: Multiple sequence alignment of MetC (Cg2536 – Accession number WP_011015029.1) with homologous sequences of *C. freiburgense* (WP_027011762.1), *C. gallinarium* (WP_191732314.1), *C. callunae* (WP_015651815.1), *C. comes* (WP_156228685.1), *C. humireducens* (WP_040086403.1), *C. pollutisoli* (NLP39668.1), *C. vitaeruminis* (WP_025253321.1), *C. rouxii* (WP_155873664.1), *C. diptheriae* (WP_014310694.1), *C. belfantii* (WP_197691522.1), *Rhodococcus* (*R. jostii* – WP_073366627.1), *Mycolicibacterium* (*M. smegmatis* – WP_233043953.1) and *Mycobacterium* (*M. tuberculosis* – WP_055353591.1) species. Clustal Omega (19) was used to create the alignment. ESPribt 3.0 (20) was used to prepare the figure. Similar residues are marked with red colored letters, completely conserved residues with a red background. Structure predictions are given as predicted by AF2 (helices are marked with squiggles, β-strands with arrows and turns with TT letters). An asterisk indicates the position mutated in the evolved strains (S322F).

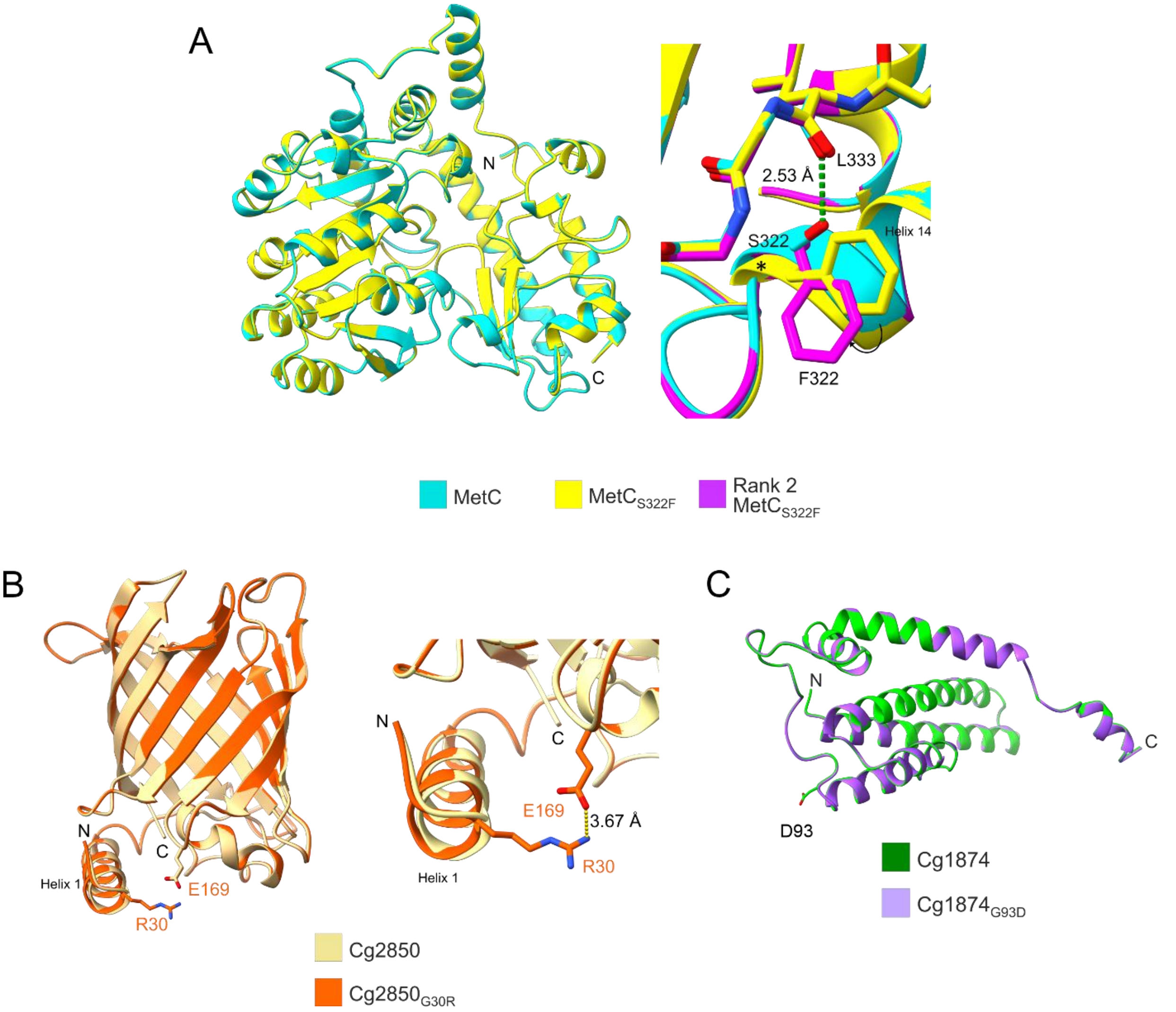

Fig. S4: AlphaFold2 models for proteins mutated during ALE of a CoNoS. (A) Left, superimposition of top-ranked AlphaFold2 predicted structures of MetC (cyan) and MetC$_{S322F}$ (yellow). Right, enlarged view of residues surrounding the site of mutation (S322F); nitrogen: blue, oxygen: red. Here, the second-rank MetC$_{S322F}$ model is included for comparison (violet). Conformational changes predicted by alternative models are indicated: a slight downward shift of the helix around the F322 position (asterisk), leaving the $\chi1$ torsion in res. 322 mostly unaffected, and a re-orientation of the F322 side chain (arrow) without major main chain displacement. The green dashed line represents the hydrogen bond formed in MetC between the S322 gamma oxygen and the L333 carbonyl oxygen. (B) Superimposition of top-ranked AlphaFold2 predicted structures (overview, left, and close-up, right) of Cg2850 (tan) and Cg2850$_{G30R}$ (orange); nitrogen: blue, oxygen: red. The sidechains R30 of the mutant and E169 of WT and mutant are displayed. The yellow dashed line represents the salt bridge between R30 and E169 of Cg2850$_{G30R}$. (C) Superimposition of AlphaFold2 predicted structures of Cg1874 (green) and Cg1874$_{G93D}$ (violet). Figure generation and structure analysis were performed using ChimeraX (21).

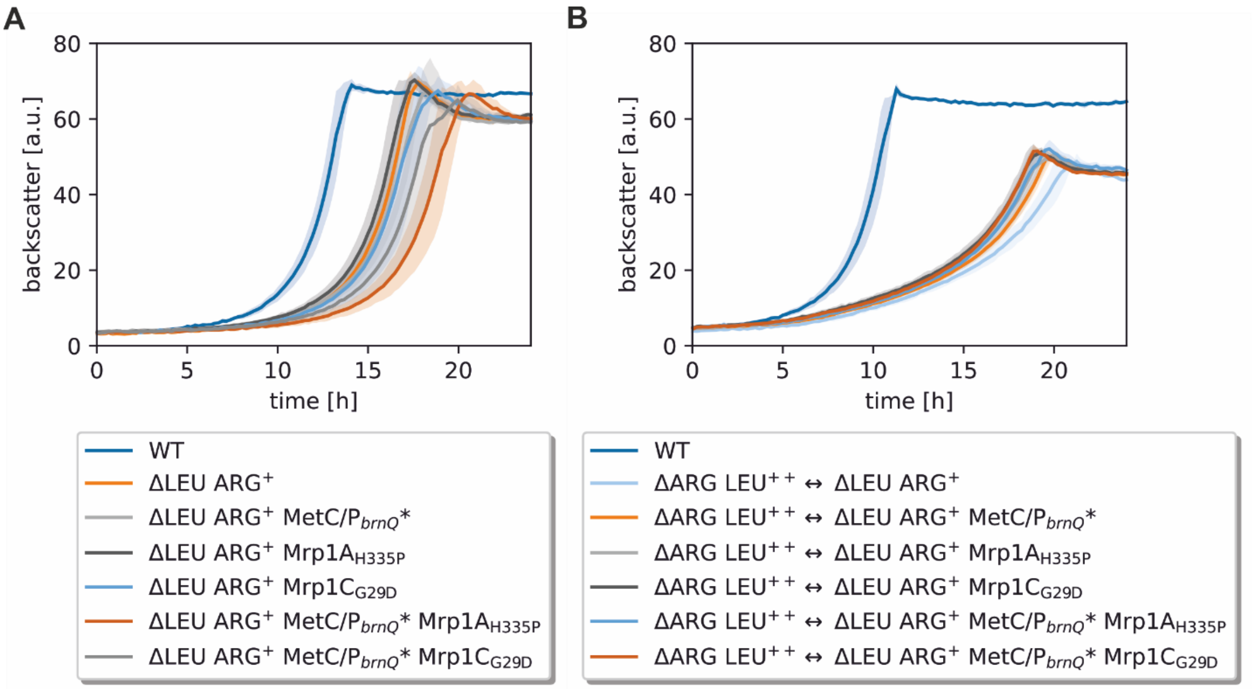

Fig. S5: Growth performance of ΔLEU ARG$^+$ reengineered strains. WT monoculture is shown as reference cultivation. (A) Monocultures of reengineered strains ΔLEU ARG$^+$ with mutations MetC/P$_{brnQ}$*, Mrp1A$_{H335P}$, and Mrp1C$_{G29D}$ as well as ΔLEU ARG$^+$ in CGXII medium with 2 % (w/v) glucose and 3 mM L-leucine. (B) CoNoS comprising one mutated strain and one parental strain in comparison with the native non-evolved CoNoS. Cultures were performed in biological triplicate in CGXII medium with 2 % (w/v) glucose. Mean values and standard deviations are shown as lines and shaded areas, respectively.

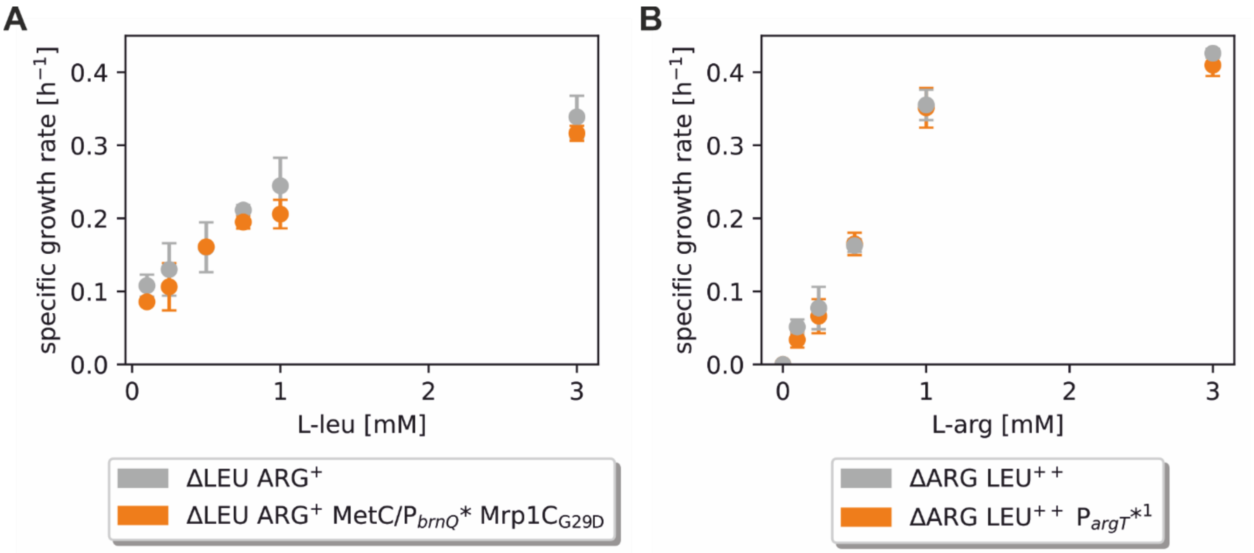

Fig. S6: (A) Growth performance of the reengineered strain ΔLEU ARG$^+$ MetC/P$_{brnQ}$* Mrp1C$_{G29D}$ and ΔLEU ARG$^+$ in monoculture supplemented with different amounts of L-leucine. Cultures were performed in biological triplicates in CGXII medium. (B) Growth performance of the reengineered strain ΔARG LEU^{++} P$_{argT}$*^{1} Mrp1C$_{G29D}$ and ΔARG LEU^{++} in monoculture supplemented with different amounts of L-leucine. Cultures were performed in biological triplicates in CGXII medium. Mean values and standard deviations are shown as points and dashes, respectively.

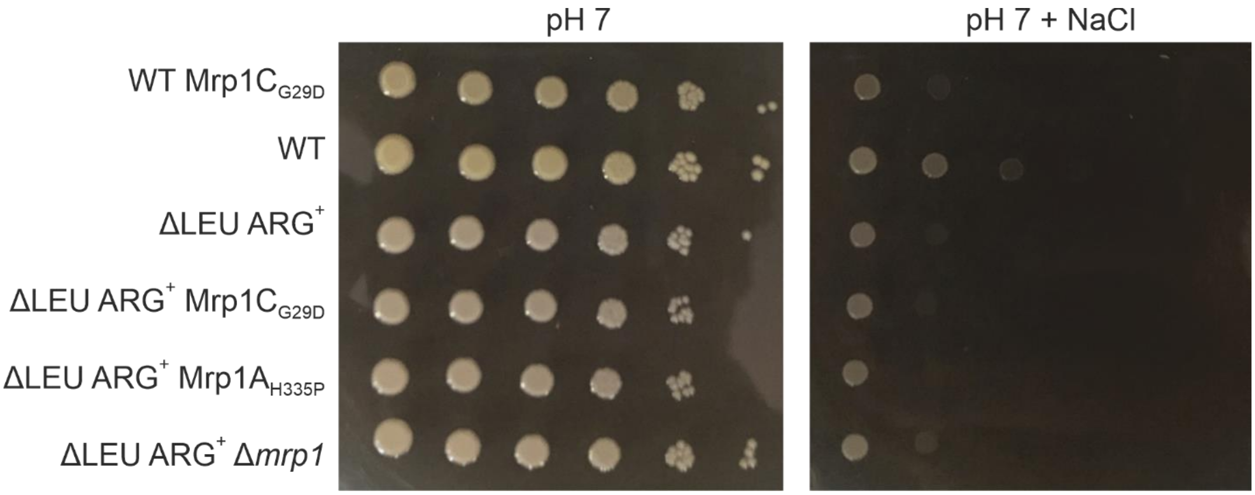

Fig. S7: Effects of Mrp1 point mutations or *mrp1* deletion on *in vivo* NaCl sensitivity of *C. glutamicum*. The given *C. glutamicum* strains were precultivated in liquid cultures first for 8 h in BHI and afterwards overnight in CGXII with 2 % (w/v) glucose and 3 mM L-leucine for auxotrophic strains. Tenfold serial dilutions were spotted onto CGXII-agar plates with 2 % (w/v) glucose, 3 mM L-leucine, pH 7 with different NaCl concentrations (left: 0 M, right 0.6 M) and incubated at 30 °C for 48 h, as performed in (22).

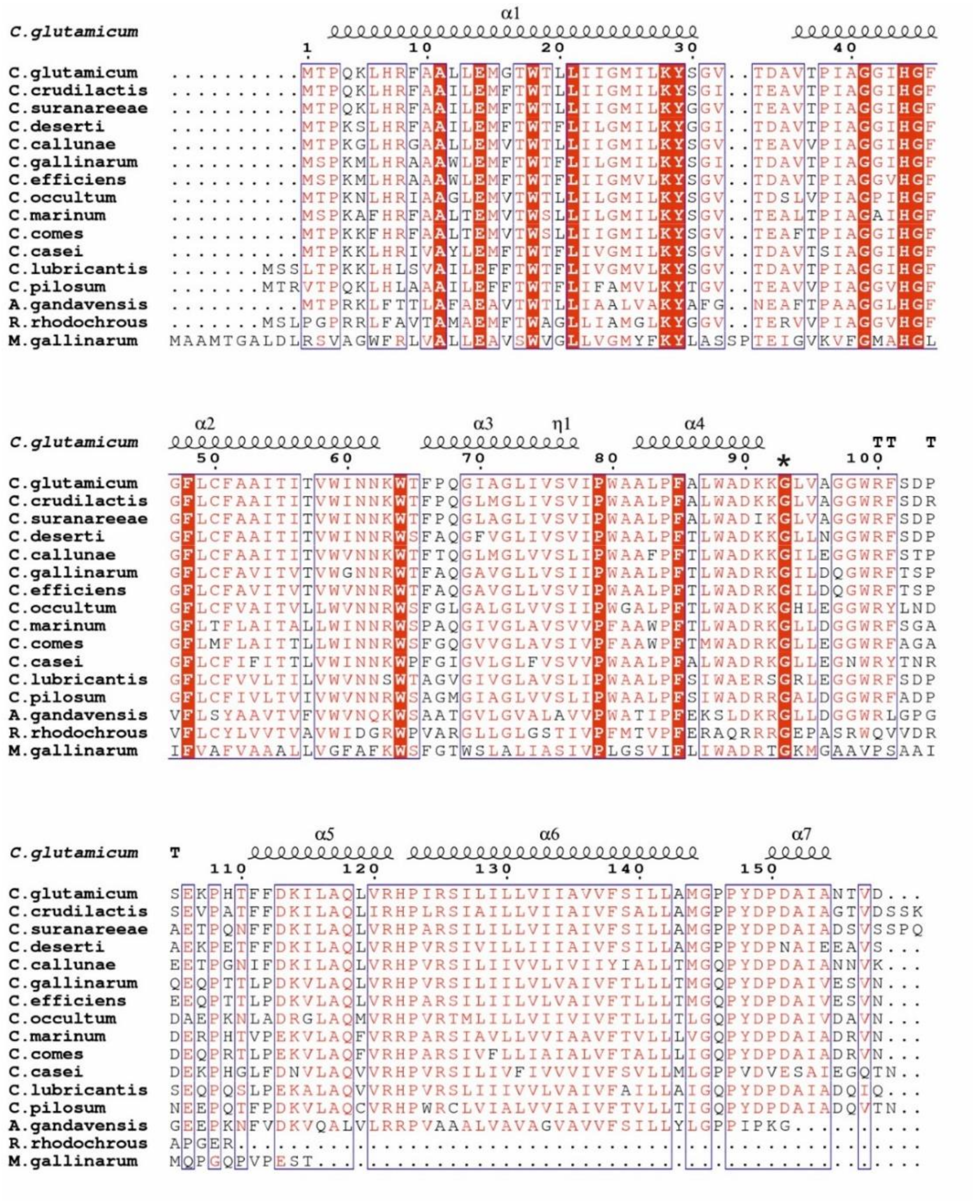

Fig. S8: Multiple sequence alignment of Cg1874 (Accession number WP_011015029.1) with homologous sequences of *C. crudilactis* (WP_066565970.1), *C. suranareeae* (WP_096456201.1), *C. deserti* (WP_053545063.1), *C. calunae* (WP_015651448.1), *C. gallinarum* (WP_191733525.1), *C. efficiens* (WP_035108832.1), *C. occultum* (WP_156231041.1), *C. marinum* (WP_042622591.1), *C. comes* (WP_156229426.1), *C. casei* (WP_006823593.1), *C. lubricantis* (WP_018295645.1), *C. pilosum* (WP_018581290.1), *Arthrobacter* (*A. gandavensis* – WP_194782106.1) *Rhodococcus* (*R. rhodochrous* – OOL31646.1) and *Mycobacterium* (*M. gallinarum* – BBY93031.1) species. Clustal Omega (19) was used to create the alignment. ESPribt 3.0 (20) was used to prepare the figure. Similar residues are marked with red colored letters, identical residues with a red background color. Structural data were assigned according to the rank 1 AF2 model (helices are marked with squiggles, β-strands with arrows and turns with TT letters). The position mutated in the evolved strains (G93D) is indicated by an asterisk.

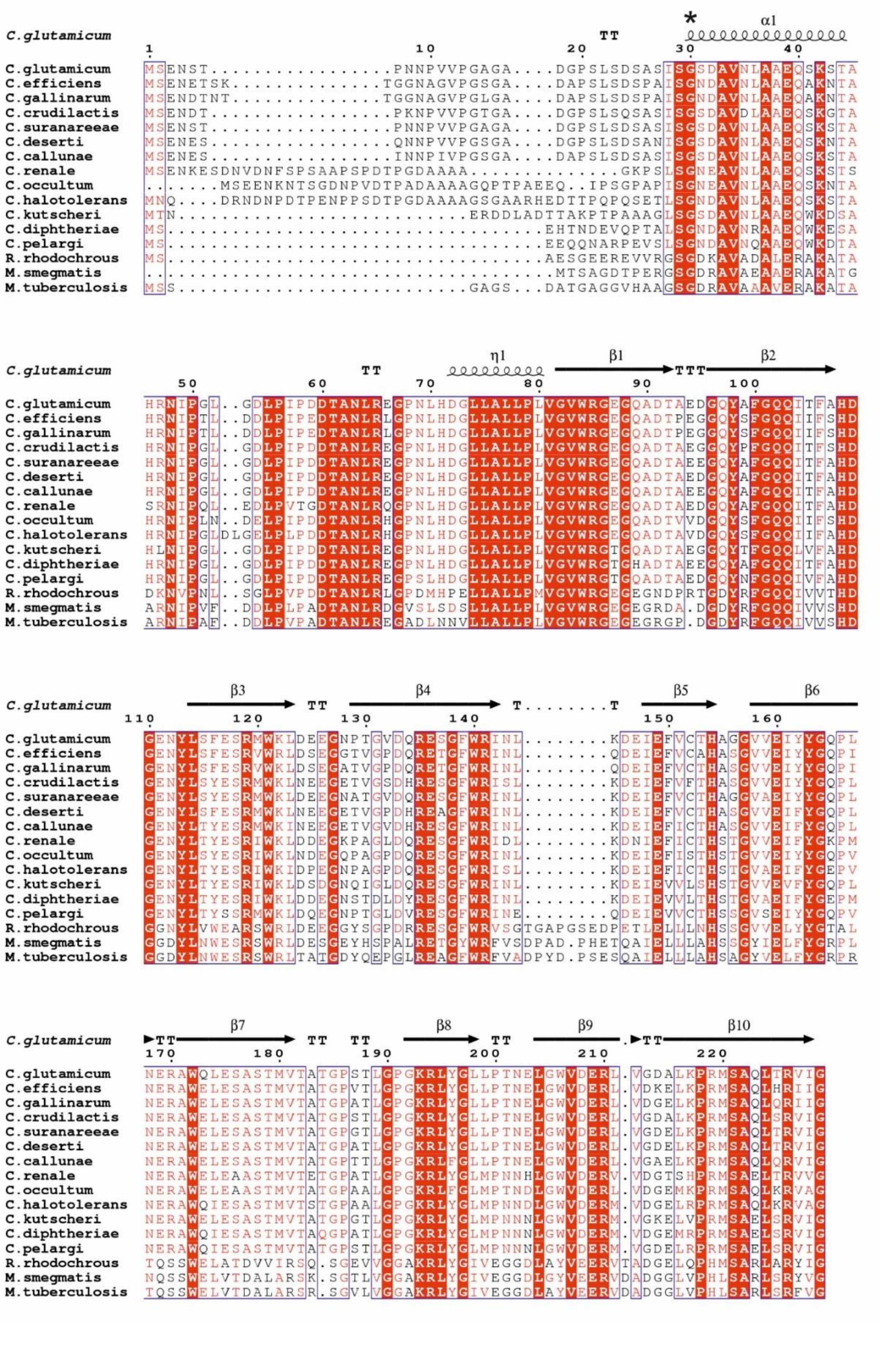

Fig. S9: Multiple sequence alignment of Cg2850 (Accession number WP_011015029.1) with homologous sequences of *C. efficiens* (WP_006769171.1), *C. gallinarum* (WP_191733242.1),

C. crudilactis (WP_066567847.1), *C. suranareeae* (WP_096458136.1), *C. deserti* (WP_053545607.1), *C. callunae* (WP_247776159.1), *C. renale* (WP_115243769.1), *C. occultum* (WP_156231609.1), *C. halotolerans* (WP_015401779.1), *C. kutscheri* (WP_046438926.1), *C. diphtheriae* (WP_014320394.1), *C. pelargi* (WP_128889270.1), *Rhodococcus* (*R. rhodochrous* – OOL29119.1) and *Mycolicibacterium* (*M. smegmatis* – WP_233043311.1) and *Mycobacterium* (*M. tuberculosis* – WP_031701164.1) species. Clustal Omega (19) was used to create the alignment. ESPribt 3.0 (20) was used to prepare the figure. Similar residues are marked with red colored letters, identical residues with a red background color. Structure predictions are given as predicted by AF2 rank 1 model (helices are marked with squiggles, β-strands with arrows and turns with TT letters). The position mutated in the evolved strains (G30R) is indicated by an asterisk.

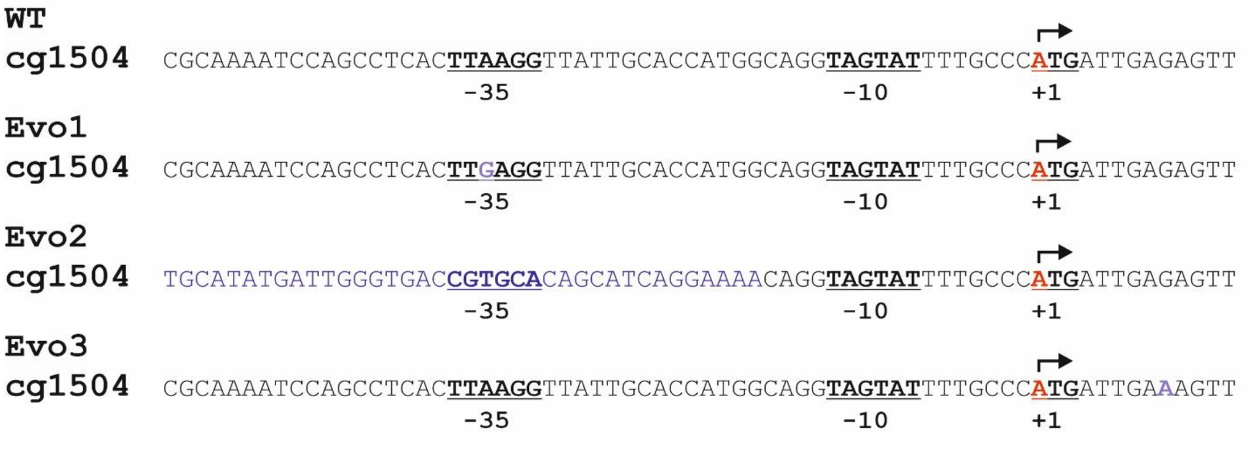

Fig. S10: Promoter regions upstream of *argT* (cg1504) in *C. glutamicum* WT and three evolved ΔARG LEU^{++} strains. -10 site according to (18), transcriptional start site (TSS) (red letter and black arrow), and mutated positions/regions in the evolved strains (purple) are given. In Evo2, 2829 bp were deleted starting 18 bp upstream of the TSS, which resulted in the given sequence.

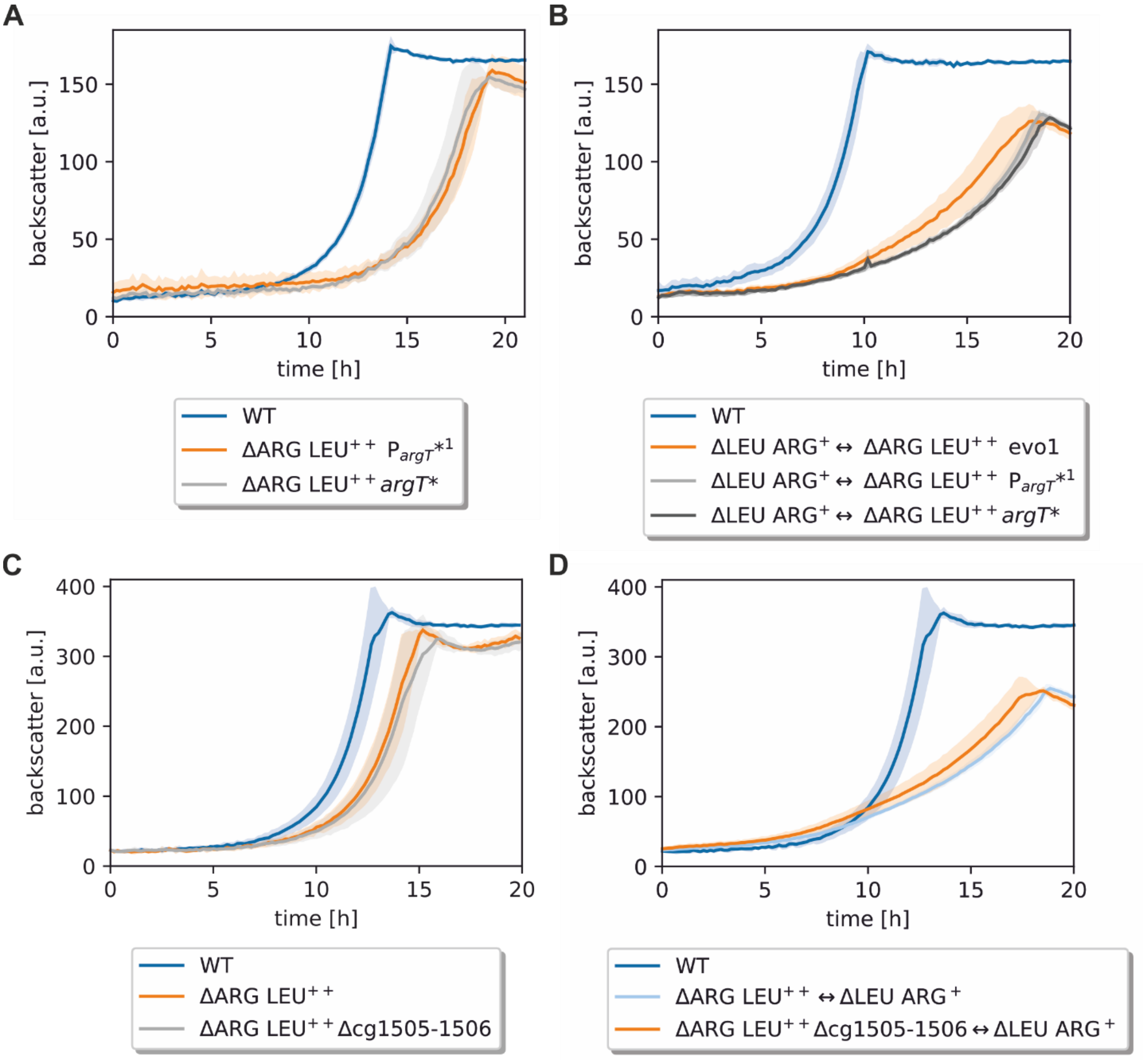

Fig. S11: Growth performance of strains with reengineered mutations in supplemented monocultures and in a CoNoS setting. Mutations were introduced into the promoter ($P_{argT}*^{1}$) or into the third codon of the coding region (*argT**). In a third strain, cg1505-cg1506 were deleted. (A) Comparison of ΔARG LEU^{++} $P_{argT}*^{1}$ and ΔARG LEU^{++} *argT** in CGXII with 2 % (w/v) glucose and 3 mM L-arginine in comparison to a WT monoculture. For comparison with the non-mutated reference strain ΔARG LEU^{++}, please refer to figure 4A where ΔARG LEU^{++} $P_{argT}*^{1}$ grew better that the reference strain. (B) Comparison of the above mentioned strains in a CoNoS setup in comparison with a CoNoS containing the evolved strain and a WT monoculture. For comparison with the non-mutated reference strain ΔARG LEU^{++}, please refer to figure 4B where the culture with ΔARG LEU^{++} $P_{argT}*^{1}$ grew better than the reference. (C) Growth of ΔARG LEU^{++} Δcg1505-1506 in comparison to ΔARG LEU^{++} and WT monoculture in CGXII medium with 2 % (w/v) glucose and 3 mM L-arginine. (D) Growth of ΔARG LEU^{++}Δcg1505-1506 in comparison to ΔARG LEU^{++} in a CoNoS in CGXII medium with 2 % (w/v) glucose. Mean values and standard deviations of biological triplicates are shown as lines and shaded areas, respectively.

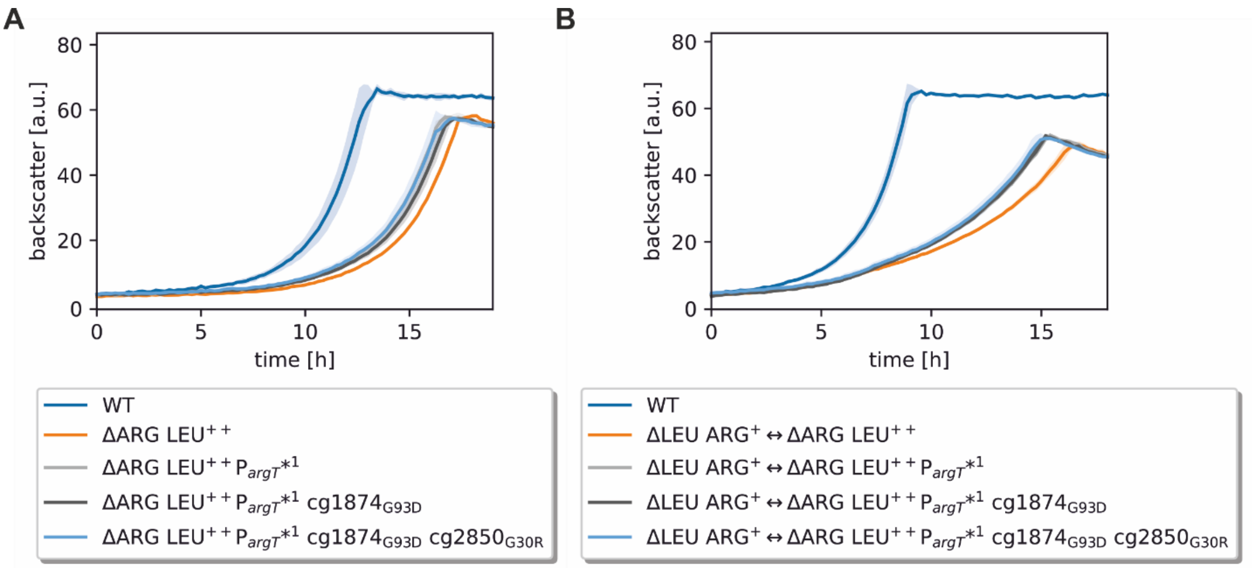

Fig. S12: Growth performance of ΔARG LEU++ strains with reengineered mutations. The WT monoculture is shown as reference cultivation. (A) Monocultures in CGXII medium with 2 % (w/v) glucose and 3 mM L-arginine. B) CoNoS cultures in CGXII minimal medium with 2 % (w/v) glucose. Mean values and standard deviations of biological triplicates are shown as lines and shaded areas, respectively.

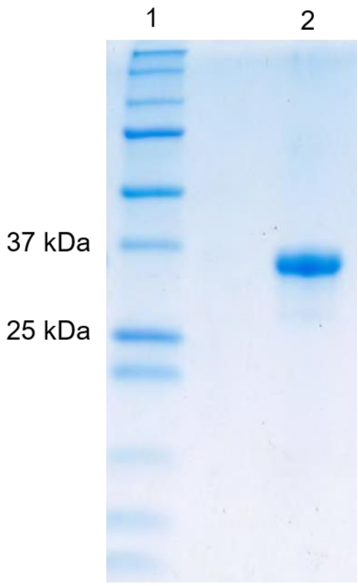

Fig. S13: SDS-PAGE of purified His$_{10}$-ArgT protein. The protein was overproduced in *E. coli* BL21(DE3) using plasmid pET-TEV-*argT* and purified by Ni-NTA affinity chromatography and subsequent size exclusion chromatography. The theoretical molecular weight of ArgT is 33 kDa. The protein sample was mixed with 5x SDS loading buffer and analyzed by SDS-PAGE and Coomassie staining. Lane 1, marker; lane 2, His$_{10}$-ArgT protein.

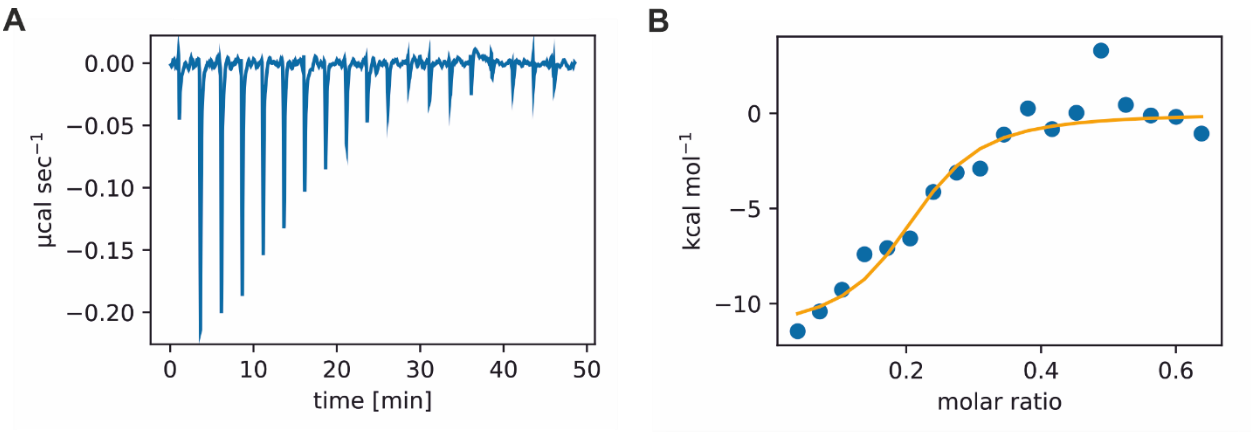

Fig. S14: Representative ITC experiment with His_{10}-ArgT and L-citrulline. (A) Raw data of a titration experiment performed with 100 µM L-citrulline and 30 µM His_{10}-ArgT in 40 mM HEPES-NaOH buffer, pH 7.4, with 100 mM NaCl. (B) Corresponding binding isotherm leading to a K_D value of 432 nM.

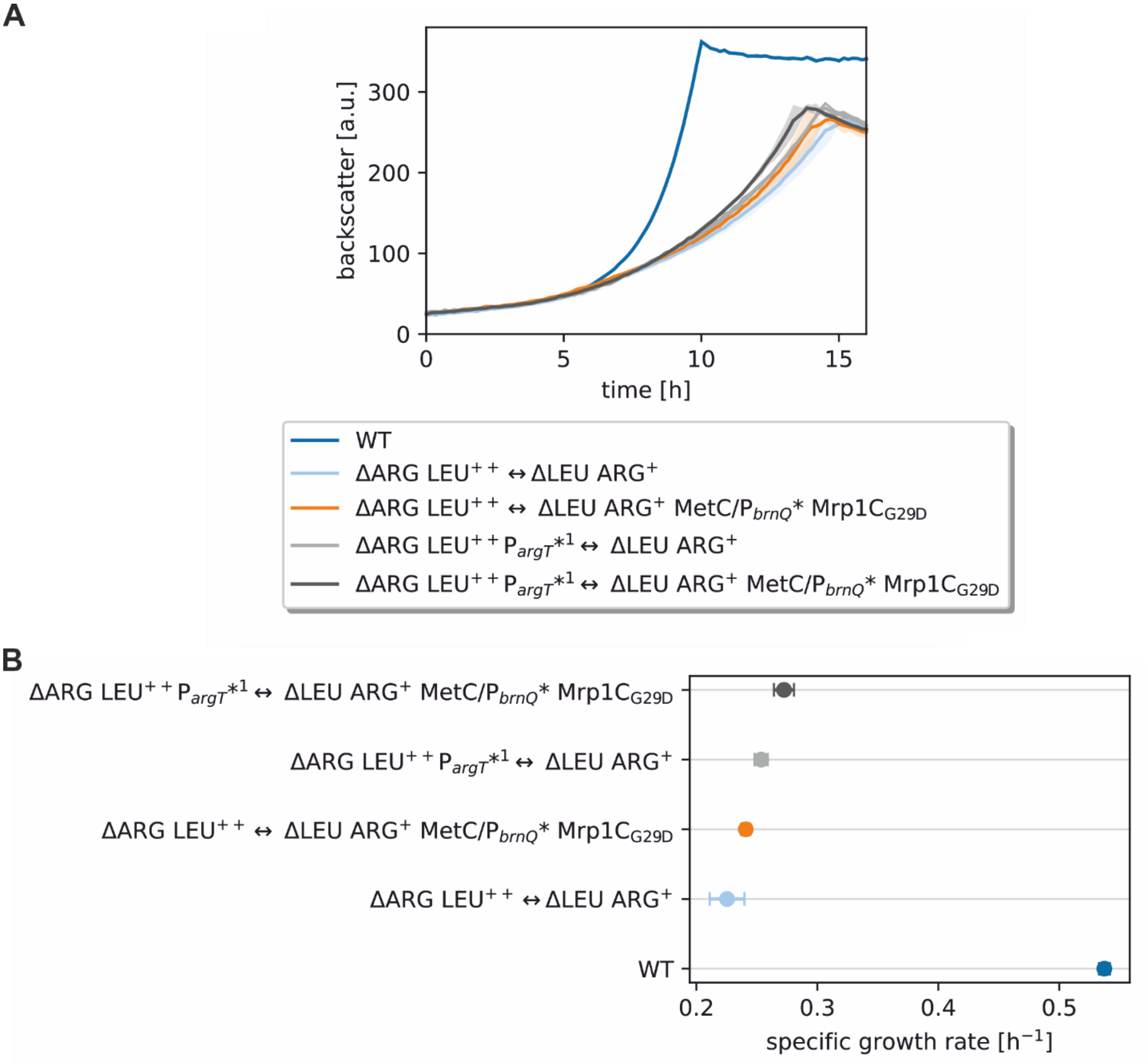

Figure S15: Growth performance of CoNoS comprising ΔLEU ARG^{+} and ΔARG LEU^{++} strains as well as these strains with reengineered mutations. Cultures were performed in biological triplicates in CGXII medium with 2 % (w/v) glucose. (A) The solid line represents the average growth of the replicates, the shady line background represents the standard deviation. WT monoculture is shown as reference cultivation. (B) Average growth rate and standard deviation of the cultures shown in (A).

References

1. Trötschel C, Follmann M, Nettekoven JA, Mohrbach T, Forrest LR, Burkovski A, et al. Methionine uptake in *Corynebacterium glutamicum* by MetQNI and by MetPS, a novel methionine and alanine importer of the NSS neurotransmitter transporter family. Biochemistry. 2008;47(48):12698-709.
2. Seep-Feldhaus AH, Kalinowski J, Pühler A. Molecular analysis of the *Corynebacterium glutamicum lysl* gene involved in lysine uptake. Mol Microbiol. 1991;5(12):2995-3005.
3. Shang X, Zhang Y, Zhang G, Chai X, Deng A, Liang Y, et al. Characterization and molecular mechanism of AroP as an aromatic amino acid and histidine transporter in *Corynebacterium glutamicum*. J Bacteriol. 2013;195(23):5334-42.
4. Wehrmann A, Morakkabati S, Krämer R, Sahm H, Eggeling L. Functional analysis of sequences adjacent to *dapE* of *Corynebacterium glutamicum* reveals the presence of *aroP*, which encodes the aromatic amino acid transporter. J Bacteriol. 1995;177(20):5991-3.
5. Zhao Z, Ding JY, Li T, Zhou NY, Liu SJ. The *ncgl1108* ($PheP_{Cg}$) gene encodes a new L-Phe transporter in *Corynebacterium glutamicum*. Appl Microbiol Biotechnol. 2011;90(6):2005-13.
6. Kulis-Horn RK, Persicke M, Kalinowski J. Histidine biosynthesis, its regulation and biotechnological application in *Corynebacterium glutamicum*. Microb Biotechnol. 2014;7(1):5-25.
7. Peter H, Bader A, Burkovski A, Lambert C, Krämer R. Isolation of the *putP* gene of *Corynebacterium glutamicum* and characterization of a low-affinity uptake system for compatible solutes. Arch Microbiol. 1997;168(2):143-51.
8. Kronemeyer W, Peekhaus N, Krämer R, Sahm H, Eggeling L. Structure of the *gluABCD* cluster encoding the glutamate uptake system of *Corynebacterium glutamicum*. J Bacteriol. 1995;177(5):1152-8.
9. Ebbighausen H, Weil B, Krämer R. Transport of branched-chain amino acids in *Corynebacterium glutamicum*. Arch Microbiol. 1989;151:238-44.
10. Tauch A, Hermann T, Burkovski A, Krämer R, Pühler A, Kalinowski J. Isoleucine uptake in *Corynebacterium glutamicum* ATCC 13032 is directed by the *brnQ* gene product. Arch Microbiol. 1998;169:303-12.
11. Peter H, Weil B, Burkovski A, Krämer R, Morbach S. *Corynebacterium glutamicum* is equipped with four secondary carriers for compatible solutes: identification, sequencing, and characterization of the proline/ectoine uptake system, ProP, and the ectoine/proline/glycine betaine carrier, EctP. J Bacteriol. 1998;180(22):6005-12.
12. Kondoh M, Hirasawa T. L-Cysteine production by metabolically engineered *Corynebacterium glutamicum*. Appl Microbiol Biotechnol. 2019;103(6):2609-19.
13. Schäfer A, Tauch A, Jäger W, Kalinowski J, Thierbach G, Pühler A. Small mobilizable multi-purpose cloning vectors derived from the *Escherichia coli* plasmids pK18 and pK19: Selection of defined deletions in the chromosome of *Corynebacterium glutamicum*. Gene. 1994;145(1):69-73.
14. Schito S, Zuchowski R, Bergen D, Strohmeier D, Wollenhaupt B, Menke P, et al. Communities of Niche-optimized Strains (CoNoS) - Design and creation of stable, genome-reduced co-cultures. Metab Eng. 2022;73:91-103.
15. Bakkes PJ, Ramp P, Bida A, Dohmen-Olma D, Bott M, Freudl R. Improved pEKEx2-derived expression vectors for tightly controlled production of recombinant proteins in *Corynebacterium glutamicum*. Plasmid. 2020;112:102540.

16. Bussmann M, Baumgart M, Bott M. RosR (Cg1324), a hydrogen peroxide-sensitive MarR-type transcriptional regulator of *Corynebacterium glutamicum*. J Biol Chem. 2010;285(38):29305-18.
17. Gibson DG, Young L, Chuang RY, Venter JC, Hutchison CA, 3rd, Smith HO. Enzymatic assembly of DNA molecules up to several hundred kilobases. Nat Methods. 2009;6(5):343-5.
18. Pfeifer-Sancar K, Mentz A, Rückert C, Kalinowski J. Comprehensive analysis of the *Corynebacterium glutamicum* transcriptome using an improved RNAseq technique. BMC Genomics. 2013;14:888.
19. Sievers F, Wilm A, Dineen D, Gibson TJ, Karplus K, Li W, et al. Fast, scalable generation of high-quality protein multiple sequence alignments using Clustal Omega. Mol Syst Biol. 2011;7:539.
20. Robert X, Gouet P. Deciphering key features in protein structures with the new ENDscript server. Nucleic Acids Res. 2014;42(Web Server issue):W320-4.
21. Pettersen EF, Goddard TD, Huang CC, Meng EC, Couch GS, Croll TI, et al. UCSF ChimeraX: Structure visualization for researchers, educators, and developers. Protein Sci. 2021;30(1):70-82.
22. Xu N, Zheng Y, Wang X, Krulwich TA, Ma Y, Liu J. The lysine 299 residue endows the multisubunit Mrp1 antiporter with dominant roles in Na^+ resistance and pH homeostasis in *Corynebacterium glutamicum*. Appl Environ Microbiol. 2018;84(10).
